# Coding of self-motion-induced and self-independent visual motion in the rat dorsomedial striatum

**DOI:** 10.1371/journal.pbio.2004712

**Published:** 2018-06-25

**Authors:** Anett J. Nagy, Yuichi Takeuchi, Antal Berényi

**Affiliations:** 1 MTA-SZTE “Momentum” Oscillatory Neuronal Networks Research Group, Department of Physiology, University of Szeged, Szeged, Hungary; 2 Neuroscience Institute, New York University, New York, New York, United States of America; EPFL, Switzerland

## Abstract

Evolutionary development of vision has provided us with the capacity to detect moving objects. Concordant shifts of visual features suggest movements of the observer, whereas discordant changes are more likely to be indicating independently moving objects, such as predators or prey. Such distinction helps us to focus attention, adapt our behavior, and adjust our motor patterns to meet behavioral challenges. However, the neural basis of distinguishing self-induced and self-independent visual motions is not clarified in unrestrained animals yet. In this study, we investigated the presence and origin of motion-related visual information in the striatum of rats, a hub of action selection and procedural memory. We found that while almost half of the neurons in the dorsomedial striatum are sensitive to visual motion congruent with locomotion (and that many of them also code for spatial location), only a small subset of them are composed of fast-firing interneurons that could also perceive self-independent visual stimuli. These latter cells receive their visual input at least partially from the secondary visual cortex (V2). This differential visual sensitivity may be an important support in adjusting behavior to salient environmental events. It emphasizes the importance of investigating visual motion perception in unrestrained animals.

## Author summary

Evolution of vision has provided us with the capacity to detect moving objects. However, visual motion perception may occur both because of the movement of an observer in an otherwise stationary environment and because of independently moving observed objects. Distinguishing these two sources or simply recognizing truly moving objects when the observer is also moving is essential for survival; it allows the reliable detection of predators or prey in real-world situations. In this study, we investigated the presence and origin of motion-related visual information in the striatum of rats, a hub of action selection and procedural memory. We found that while many of the striatal neurons responded to the apparent visual motion while the rat was running in an environment with striped walls, only a subset of them could also respond to moving stripes with similar properties if watched passively. This differential visual sensitivity may be an important mechanism of adjusting behavior to salient environmental events. The need for congruent locomotion to evoke neuronal responses in many of the recorded neurons emphasizes the importance of investigating visual motion perception in unrestrained animals.

## Introduction

Besides the well-known motor functions of the basal ganglia [1], there is massive evidence for its role in higher-order integrative functions. While the ventral part of the caudate putamen (CPu) is mainly associated with reward and motivation-related processes (e.g., reinforcement [2]), the dorsal part is involved in executive functions based on both stimulus response learning [3] and procedural memory [4–6]. Neurons of this dorsal sensorimotor striatum can perceive elementary visual cues representing luminance changes [7,8] and visual motion [9–12] and integrate them with other modalities [13,14] to serve their function in multiple potential ways. On one hand, the self-motion-induced changes in the visual world (i.e., due to the dislocation of the observer) would serve as feedback for both corollary discharge [15–17] and for refining motor outputs [18]. On the other hand, the detection of the self-motion-independent object motion in an otherwise stationary environment would be very important for recognizing approaching predators and other threats or escaping prey. This latter process may be important for survival by facilitating appropriate action selection through perceptual decision-making [19].

Previous experiments that studied the motion sensitivity of the CPu at the single-neuron level employed anesthetized or restrained animals. Thus, they were not designed for deciphering if the CPu is equally sensitive for the self-motion-induced (i.e., dislocations of the visual scene congruent with self-motion) and the self-motion-independent object motion. More profoundly, there is no existing evidence that the CPu neurons can distinguish between these two conditions at all nor whether they can integrate the visual motion percepts with spatial representations or other aspects of striatal information processing.

To answer this question, we investigated the response profiles of the dorsal CPu neurons to self-independent and self-motion-induced visual motion stimuli in freely moving rats. We did this by conducting large-scale, high-density extracellular recordings. We also made efforts to show the role of the corticostriatal pathway in shaping these responses, as there is no consensus regarding the origin of the striatal visual information either [7,20–22].

## Results

**Fig 1:**
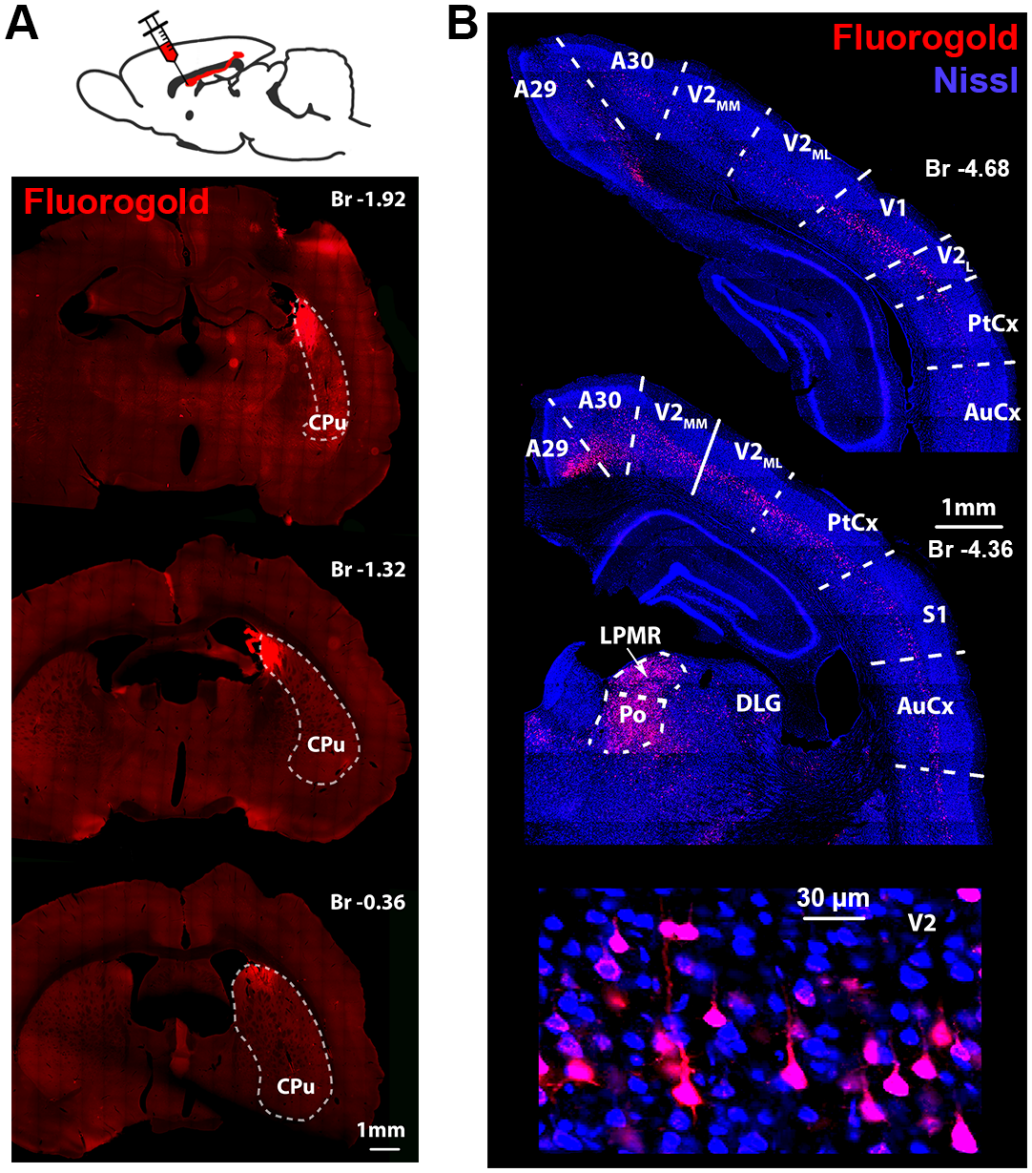
Anatomical connectivity of the dorsomedial striatum. (A) Injection schematic and representative micrographic images of the striatal injection site of the retrograde tracer Fluorogold. (B) Retrogradely labeled cell bodies were found in the V1 and V2 ipsilaterally, as well as in the posterior (“Po”) and LPMR thalamus. Note the lack of labeling at the DLG. Bottom panel shows a magnified image of the area V2_ML_ of the middle panel. The thick apical dendrites and the location of the somata suggest that the labeled neurons are putative pyramidal neurons. AuCx, auditory cortex; Br, bregma; CPu, caudate putamen; DLG, dorsolateral geniculate nucleus; LPMR, lateral-posteromedial; V1, primary visual cortex; V2, secondary visual cortex; V2_L_, lateral secondary visual cortex; V2_ML_, mediolateral secondary visual cortex; V2_MM_, mediomedial secondary visual cortex.

We conducted our experiments on male Long-Evans rats (*N* = 32, 285–655 g; 3–12 mo old). The intermediate part of the dorsomedial striatum has been reported to display visual stimulus—induced electrophysiological responsivity in many species [7,9–11]. This region has also been reported to receive axons from both the A18 and A18b secondary visual cortical areas and to be the exclusive striatal recipient of innervation from the primary visual cortex (V1) [20,23]. To confirm this finding, we injected FluoroGold to the dorsomedial CPu (*N* = 4 rats, Fig 1A) and identified regions with retrogradely labeled neuronal somata. Similarly to previous results obtained in mice and rats [7,23], we found that neurons of the secondary visual cortex (V2, and also the most rostral part of the V1) send axonal projections to the injected area of the CPu (Fig 1B). Based on their large somata, their thick apical dendrites, and their restricted layer V locations, the labeled neurons are putative pyramidal neurons. We did not find any labeled neurons at the more caudal parts of the V1 or in the lateral geniculate nucleus, but the lateral-posteromedial and posterior nuclei of the thalamus were densely labeled.

**Fig 2:**
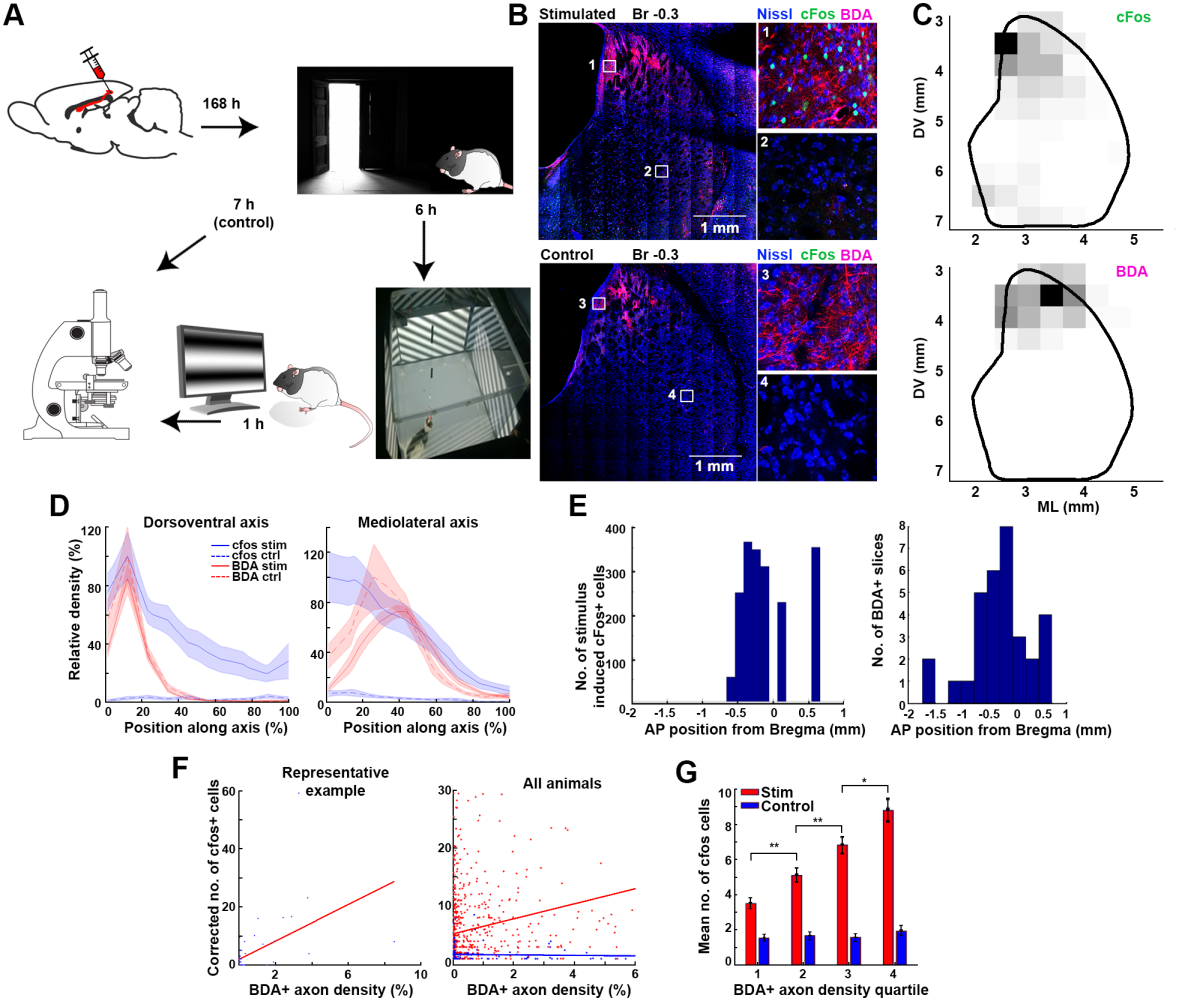
Colocalization of the stimulus-induced striatal cFos expression and the axon terminals from the visual cortex. (A) Experimental schematics. (B) Representative example sections of a visually stimulated (upper panels) and a control animal (lower panels). Blue, red, and green color channels are assigned to the respective fluorochromes of the Fluoro Nissl, BDA, and cFos immunohistochemistries. Insets show the magnified areas marked by rectangles on the left panels. Note the lack of cFos+ neurons on the lower (control) panels, while the anterograde BDA tract tracing from the visual cortex shows a similar axonal distribution pattern in both cases. (C) Unsupervised quantification of the striatal distribution of cFos+ neurons (top panel) and BDA+ axons (bottom panel) in a representative coronal section of a stimulated animal’s brain. See also S1 Fig. (D) cFos (blue) and BDA (red) density distribution in stimulated (solid lines) and control animals (striped lines). Left and right panels show the distributions along the respective DV and ML axes of the striatum. (E) AP distribution of visual stimulation—induced cFos+ somata (left panel) and the distribution of slices with detectable FluoroGold-labeled axons (*N* = 37 slices in 9 animals). Note that the BDA+ axons target the similar AP segment of the CPu where the visually inducible cFos activity was present. (F) Correlation of the normalized cFos and BDA densities in a representative stimulated animal (left panel) and in all animals (right panel). The right and blue dots of the right panel and regression lines, respectively, represent data from the stimulated and sham animals. (G) Mean number of the cFos+ neurons in the striatum calculated for the 4 BDA axon density quartiles. Note that while the higher BDA density predicts higher cFos+ cell numbers in the stimulated animals (red), the cFos+ density is constant with no respect to the BDA distribution in the control animals (blue). AP, anteroposterior; BDA, biotinylated dextran amine; CPu, caudate putamen; DV, dorsoventral; ML, mediolateral.

To evaluate the colocalization of the axons originating from the visual cortex and the visually active CPu neurons on the fine scale, we injected biotinylated dextran amine (BDA) at the V2 locations identified in our retrograde tracing experiment (Fig 2A). After 1 wk of survival, the animals were placed into a 60 × 60 cm large box whose 35 cm tall walls featured 4 liquid crystal display (LCD) monitors to provide a self-motion-independent, passive visual (pVis) stimulation. For the initial 6 h, the monitors were turned off, and the animals were kept in darkness. Then, a pseudorandom sequence of moving visual gratings and blinking lights was displayed on the monitors for 1 h while the animals were randomly exploring the box (Fig 2A, for details of the pVis stimulation, see the Materials and methods section). The control animals were kept in darkness for 7 h without any stimulation. The rats were overanesthetized and perfused. Coronal sections of their brains were developed to visualize BDA-positive axons and the expression of the cFos (Fig 2B). We also tested if the cFos+ neurons were parvalbumin positive (PV+). We found that the presence of the two markers were almost mutually exclusive (as demonstrated by the anti-cFos and anti-PV double immunostaining in 4 sections of 1 rat, *N* = 490 and 406 single positives for anti-cFos and anti-PV, respectively, and only 3 double-positive neurons).

Automated, unsupervised algorithms were used to quantify the BDA+ axon density and the number of cFos+ neurons in 425 × 425 μm squares in each slice of the CPu (Fig 2C and S1 Fig). We found that axons originating from V2 neurons colocalized with the cFos+ neurons. Both the BDA+ and cFos+ locations were restricted to the dorsal part of the CPu. Regarding the mediolateral (ML) extent, cFos activity was present at the medial and intermediate locations, while BDA was mainly present at the intermediate (Fig 2D). The induced striatal cFos expression was present only between AP coordinates −0.7 and 0.7 from the bregma, and V2 axons targeted the same intermediate segment of the CPu between anteroposterior (AP) coordinates −1.7 and +0.5 mm from the bregma (Fig 2E).

The BDA+ axon densities in the coronal plane were spatially correlating with the cFos+ density maps of the stimulated animals (stimulated: R = 0.20, *p* < 0.005, *N* = 696 density pairs from 5 animals; control: R = −0.03, *p* = 0.69, *N* = 134 density pairs from 4 animals, Pearson’s linear correlation, Fig 2F). We concluded that the cFos expression was a result of the visual stimulation, as it was uniformly low at every location in the control animals (stimulated: 3.51 ± 0.30, 5.12 ± 0.42, 6.84 ± 0.50, 8.82 ± 0.66 cFos+ neurons found at consecutive BDA density quartiles, *p* = 0.002, 0.009, 0.019 for interquartile comparison, *N* = 4 × 174 density values; control: 1.55 ± 0.19, 1.69 ± 0.19, 1.59 ± 0.22, 1.94 ± 0.28 cFos+ neurons found at consecutive BDA density quartiles, *p* = 0.61, 0.72, 0.33 for interquartile comparison, *N* = 4 × 34 density values; two-sample *t* test; Fig 2G).

Next, to delineate the functional details of the visual responsivity of the CPu neurons, we conducted electrophysiological investigations on the rostrocaudal segment of the CPu that we previously identified. We recorded the activity of a total of 734 neurons using silicon electrodes (“silicon probes”) in freely moving rats (*N* = 42 sessions in 6 animals) performing various behavioral and visual stimulation tasks. The recorded single units were isolated from the compound electrical activity based on their action potential waveforms, using a semiautomated clustering method [24,25]. Noisy units or multiunits were discarded, while clusters meeting our predefined isolation quality criteria [26] were classified as phasically firing neurons (PFNs, *N* = 467, 63.62% of all isolated single units), fast-firing neurons (FFNs, *N* = 163, 22.21%), and tonically firing neurons (TFNs, *N* = 56, 7.63%). These classifications were based on the electrophysiological features of the corresponding waveforms and spike trains. These classes putatively match the morphological and neurochemical groups of medium spiny, PV+ aspiny, and cholinergic aspiny neurons, respectively [4,27] (S2 Fig). In 6% of the recorded single units, the classification was ambiguous because of their very low firing rate (FR). Those units were discarded from further analysis.

**Fig 3:**
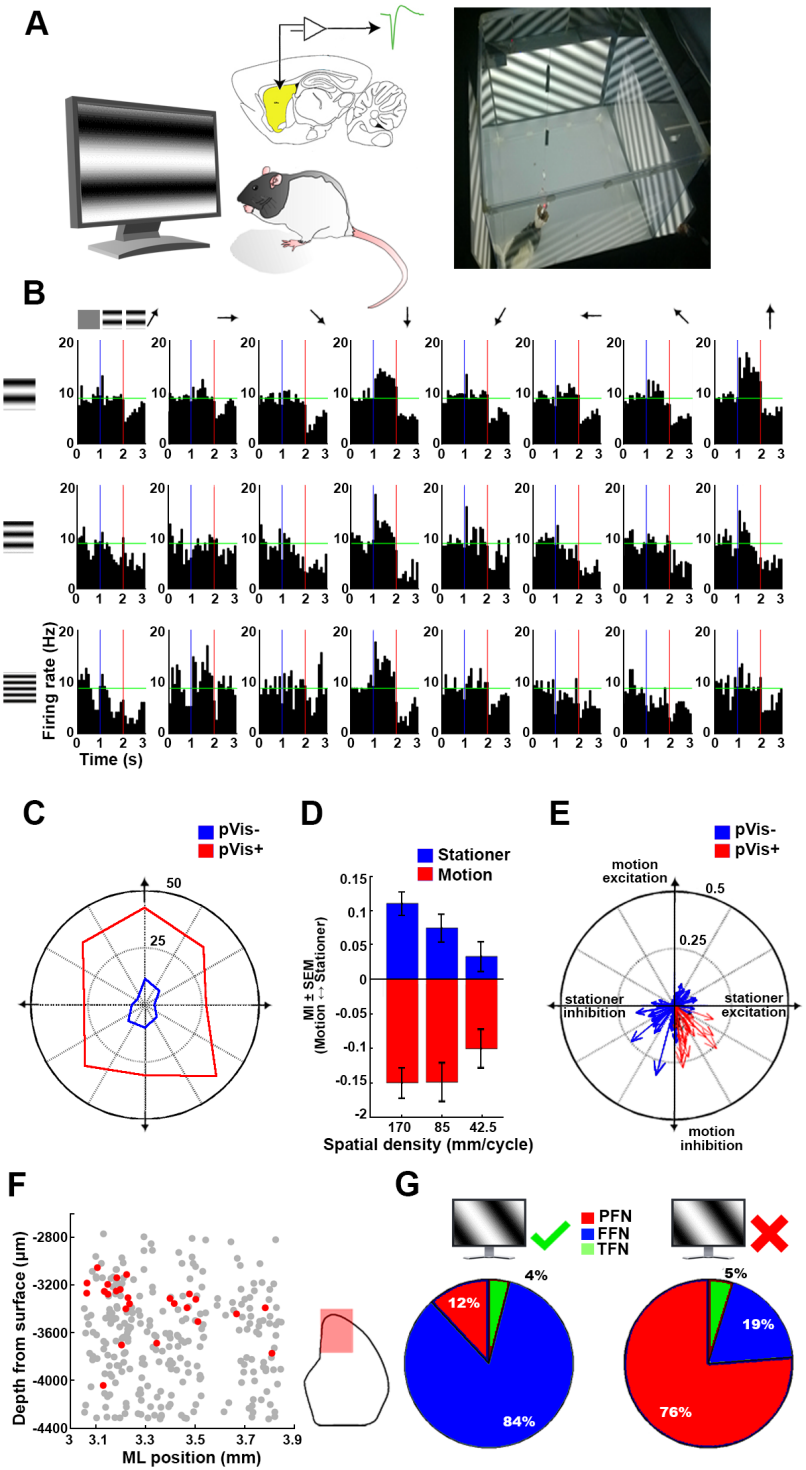
Self-motion-independent pVis stimulation of the striatal neurons. (A) Schematics of the experimental arrangement. The striatum is marked by yellow shading. (B) Peristimulus time histograms of a pVis+ striatal neuron’s responses during visual stimulation using 3 × 8 parameter combinations. The first third of each peristimulus time histogram represents the activity of the neuron while watching a uniform gray image. The middle and right thirds, respectively, reflect the responses during still and moving stripes. Horizontal green lines represent the spontaneous FR of the same neuron. Note that horizontally oriented still patterns evoked increased FRs, while moving stripes caused inhibition with no respect to the direction of the motion. (C) Rose plot showing the percent of pVis+ and pVis− neurons responsive to the 8 different directions tested. The axes of the plot match the movement directions. Note that neither the pVis+ (red) nor the pVis− (blue) neurons expressed any obvious preference toward any motion directions. (D) Mean MIs of all pVis+ neurons during stationary (blue bars) and moving stimulation (red bars). The pVis+ neurons were more sensitive to low spatial and high temporal frequencies during both still and moving stimulation. (E) Response vectors of pVis+ (red) and pVis− (blue) neurons. The MIs of the 24 stimulus conditions are averaged for each cell. Still and moving MIs are assigned to the X and Y components of each response vector. Note that all pVis+ neurons expressed stationary stimulus-induced excitation combined with motion-induced inhibition. (F) Location of the pVis+ neurons (red dots) in the coronal plane. Gray dots denote the location of the nonresponsive neurons. The red-shaded part of the inset on the right shows the area covered by the electrophysiological investigations. Note that the visually responsive cells were mainly present at the dorsomedial segment of the recorded area. (G) Distribution of cell types among the pVis+ (left) and pVis− (right) neurons. The majority of the visually excitable neurons were FFNs, while the distribution of the other neurons followed histological proportions. FFN, fast-firing neuron; FR, firing rate; MI, modulation index; ML, mediolateral; PFN, phasically firing neuron; pVis, passive visual; TFN, tonically firing neuron.

To test the responsivity of the CPu neurons to moving visual stimuli, animals were placed into the visual stimulation box used in the cFos expression experiments (Fig 3A). Each stimulation trial began with presenting a nonpatterned gray background for 500 ms (uniform screen), followed by 500 ms of sinusoidally modulated stationary grating pattern (stationary grating). Lastly, the pattern was gradually moved to be perpendicular to the grating’s axis (moving grating) for another 500 ms. The spatial density of the grating, the velocity, and the direction of the motion were all varied in a pseudorandom order until each parameter combination was repeated at least 150 times [9,28,29]. Neurons were considered responsive if they significantly altered their FR in response to either the stationary or the moving grating for at least 2 of the parameter combinations. Altogether, 289 neurons were tested using this paradigm (21 sessions of pVis stimulation), and only 25 of them were considered responsive to the self-motion-independent pVis stimulation (pVis+ neurons). In general, we found no systematic preference toward any of the tested directions of motion. Some neurons responded similarly to all directions, while others preferred only a few adjacent directions or two opposing ones (Fig 3B and 3C).

Regarding the spatial resolution of the grating and the velocity of the motion, neurons were clearly more sensitive to broader stripes sliding with a high velocity compared to the dense, slowly shifting gratings (*p* < 0.005 for all comparisons except thick versus medium moving stripes, for which *p* = 0.92; *N* = 200 in each group; paired *t* test; Fig 3D). To describe the general responsiveness of these neurons, we averaged the modulation indices (MIs) of the stationary and moving conditions (calculated against the uniform condition) across all stimulus conditions. Surprisingly, the response combinations occupied only a restricted subset of the available state space: the pVis+ neurons either increased their firing in the presence of the stationary grating or became inhibited by the moving gratings or from the combination of these two response types (Fig 3E). The pVis+ neurons were not uniformly distributed among the recorded spatial locations. They were mainly located at the mediodorsal part of the CPu (Fig 3F). The majority were FFNs (84%; 12% PFNs; and only 1 neuron was classified as a TFN). In contrast, the distribution of the putative cell types among pVis− neurons resembled the histological proportions of these classes (76% PFN, 19% FFN, and 5% TFN) (Fig 3G).

**Fig 4:**
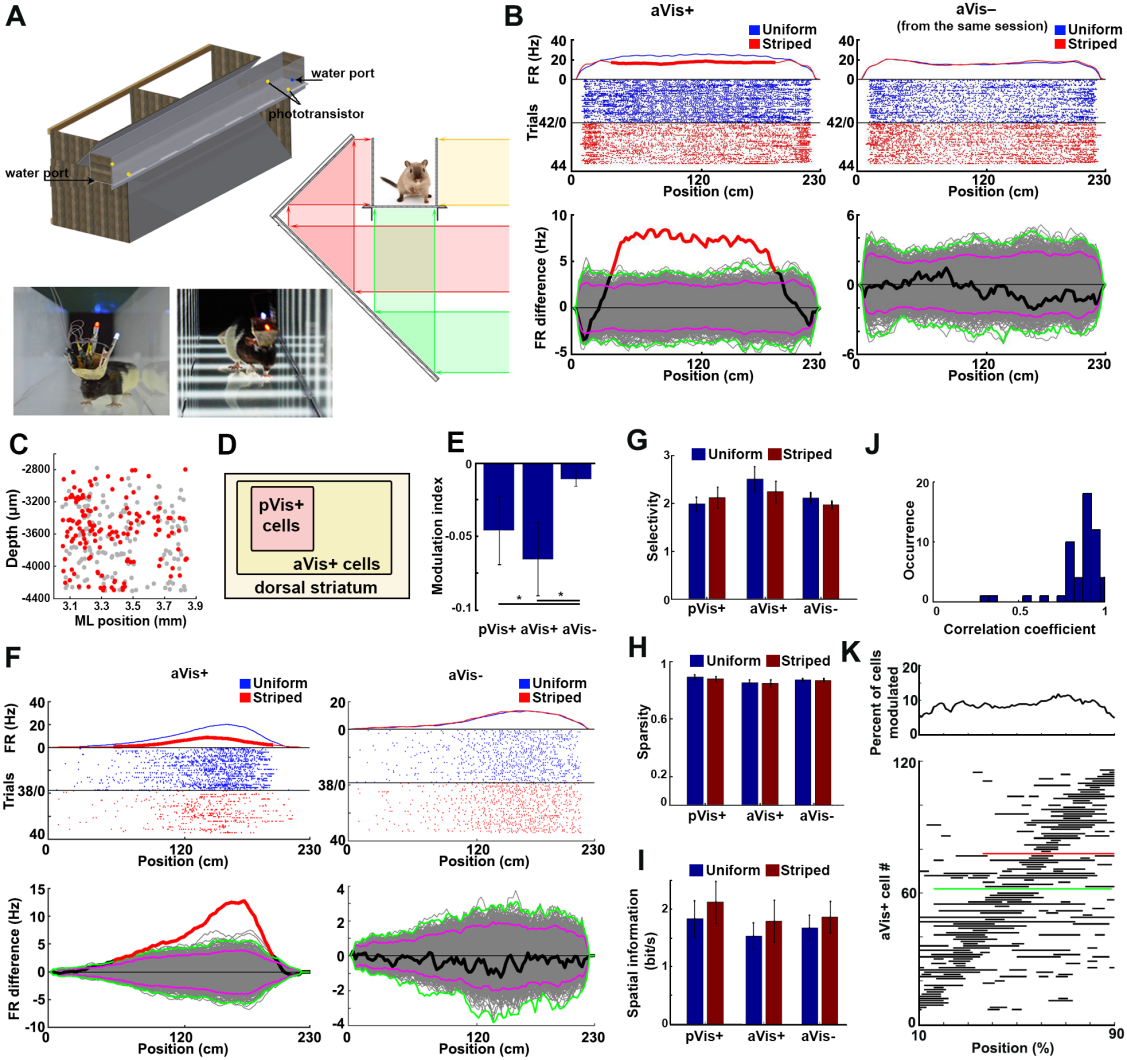
Self-motion-induced aVis stimulation of the striatal neurons. (A) Schematics (left) and cross section (middle) of the linear maze with a dynamically updated visual environment. The bottom panels show photographs of rats running in the uniform and the striped environment. (B) Raster plots and place-resolved FRs of a representative pVis+/aVis+ (left, called as pVis+) and an aVis−/pVis− (right, called as aVis−) neuron simultaneously recorded during the same session. The blue dots and curve represent trials during uniform maze running; the red ones show the firing patterns in the striped maze. Note that the two conditions were randomly alternated from trial to trial. For display purposes, trials are reordered by the stripedness. Lower panels show the details of the statistical tests of space-resolved visual modulation. The thick black line shows the real FR difference, while the gray curves denote the label-shuffled surrogate differences. Purple and green lines depict the “local” and “global” significance thresholds [30], respectively. Spatial locations breaking the global significance limits are marked with a thick red line. (C) Location of the aVis+ neurons (red dots) in the coronal plane. The gray dots denote the location of the nonresponsive aVis− neurons. aVis+ neurons were distributed more uniformly in the recorded area than the pVis+ cells. (D) Venn diagram of the CPu neurons showing the set relationship of the aVis+ and pVis+ neurons. Note that all recorded pVis+ neurons were also aVis+ at the same time. (E) Mean modulation indices of pVis+, aVis+, and aVis− neurons in the aVis environment. Note that pVis+ neurons decreased their FRs when the rat was running in the striped maze, similar to their behavior in the pVis experiment. (F) Two representative striatal neurons with place field—like properties recorded simultaneously in an aVis session. Note that the activity of the left neuron was rate modulated by the stripedness (an aVis+ neuron), while the right example neuron was showing an almost identical spatial response profile in both the uniform and the striped conditions (aVis− neuron). Bottom panels show the details of the space-resolved statistical tests as on panel B. (G, H, I) Spatial coding efficiency expressed as “spatial selectivity” (G), “sparsity” (H), and “spatial information” (I) of the recorded neurons in uniform (blue bars) and striped conditions (red bars). (J) Distribution of the correlation coefficients of the spatially binned FRs of neurons with place field—like activity during running in the uniform and the striped environments. The place fields were stable across both the uniform and the striped conditions, as expressed by the high spatial correlation values of the neurons with place field—like activity. See also S5 Fig. (K) Distribution of the spatial segments at which the FRs of the aVis+ neurons were significantly rate modulated by the perceived visual motion (bottom). Green and red lines represent the example aVis+ neurons shown on panels “B” and “F”, respectively. Note that the significantly modulated segments uniformly tiled the whole extent of the maze (top plot). aVis, active visual; FR, firing rate; ML, mediolateral; pVis, passive visual.

The previously mentioned moving visual stimuli were presented while the animals were either standing still or when their movements were random compared to the perceived intensive visual motion (which resembled self-independent object motion). These pVis stimuli may be processed differently from the self-motion-induced optic flow of natural scenes, which is concordant with the other senses (e.g., the proprioception); thus, we decided to also investigate the visual responsivity of CPu neurons during self-motion. In contrast to experiments on head-fixed animals placed in a virtual environment, our aim was to generate a realistic visual experience that was congruent with the movements of the animals (active visual [aVis] stimulation), in order to preserve all the proprioceptive, vestibular, and exteroceptive aspects of the movements. We trained the animals to run in a linear maze back and forth for a water reward. To keep control of the visual environment seen by the rats during each run, we built the walls of the linear maze from translucent plexiglass. We placed it in a frame equipped with 2 perpendicular mirrors along the whole track (Fig 4A). Computer-generated visual scenes were projected by an LCD projector onto both the walls and the floor of the linear maze, with the help of mirrors to avoid shading by the rat. Before each trial, the projected scene was changed to either a uniformly gray (“uniform maze”) or a grated pattern with similar spatial frequency and luminance as was used during the pVis stimulation (“striped maze”) (Fig 4A). The “uniform” and “striped” patterns were isoluminant, and they were pseudorandomly alternated trial by trial to avoid the biasing effect of slow changes in internal brain dynamics.

In total, 685 CPu neurons were tested in this task (in 41 sessions with 6 rats, including the neurons also tested in the pVis environment). In marked contrast to the small proportion of pVis+ neurons, 325 of them (62% PFN, 26% FFN, and 11% TFN) displayed a significant change in their FRs in response to the striped environment (aVis+ neurons). Fig 4B shows a representative example of an aVis+ (FR = 0.80 ± 0.10 Hz and 0.56 ± 0.08 Hz in the respective uniform and striped trials, *p* < 0.01, paired *t* test, *N* = 20 spatially binned FR pairs; more examples are shown on S3 Fig) and an aVis− neuron (FR = 0.53 ± 0.08 and 0.53 ± 0.1 Hz in the respective uniform and striped trials, *p* = 0.76) recorded simultaneously with its aVis+ peer. The aVis+ neurons were not restricted to the most superficial and medial part of the mediodorsal CPu. Instead, they were distributed more uniformly (Fig 4C).

Importantly, all pVis+ neurons were also responsive in this task. Thus, the pVis+ cells can be considered as a subgroup of the aVis+ neurons (Fig 4D). For simplicity, from here on we will refer to the double responsive (i.e., pVis+/aVis+) neurons as pVis+ neurons, and those cells which were only responsive in the aVis environment but not in the pVis one (i.e., pVis−/aVis+ neurons) as aVis+ neurons.

The firing pattern of the pVis+ neurons in the uniform maze was more deterministic with regard to their activity in the striped maze, compared to the pVis− neurons (R = 0.77 ± 0.02 and 0.65 ± 0.02 for pVis+ and pVis− neurons, respectively; Pearson’s linear correlation between uniform and striped runs, *p* = 0.032, two-sample *t* test, S4 Fig). In agreement with their behavior during the pVis stimulation experiment, the pVis+ neurons decreased their FRs while the rats were running in the striped linear maze.

The visual modulation of the aVis+ neurons in this task was similar to the response profiles of the pVis+ neurons (*p* = 0.047 for pVis+ versus aVis−, and 0.041 for aVis+ versus aVis− comparisons, MI = −4.7% ± 2.3%, −6.9% ± 2.7%, and −1.0% ± 0.5%; *N* = 12, 133, and 50, respectively, for the pVis+, aVis+, and aVis− neurons, two-sample Kolmogorov-Smirnov test; Fig 4E). This result confirmed the visual motion-induced inhibition observed in the passive stimulation task.

The dorsomedial part of the intermediate striatum is known to integrate highly heterogeneous, multimodal inputs in mice [31]. We were therefore interested if pVis+ and/or aVis+ neurons also code context- or location-dependent features as suggested by earlier studies [32,33]. A significant number of neurons showed spatial location—selective activity, similar to the place cells [33,34] of the hippocampal formation (36% of the pVis+, 20% of the aVis+, and 17% of the aVis− neurons). All of these neurons had one single place field similar to the proximal CA1 and CA3c regions of the hippocampus [35]. However, their “place fields” were relatively large, usually spanning over more than one-third of the track (Fig 4F and S3 Fig).

The FRs of these “place cell—like” neurons were rate modulated by the presence of striped patterns to a similar extent as the activity of those that were active at every location. The spatial coding ability of the aVis+ neurons was slightly (but not significantly) better than either the pVis+ or the aVis− neurons and was not influenced by the presence of the striped pattern (selectivity of pVis+ cells: 1.98 ± 0.14 and 2.11 ± 0.21; aVis+ cells: 2.46 ± 0.3 and 2.33 ± 0.28; aVis− cells: 2.11 ± 0.11 and 1.96 ± 0.09; sparsity of pVis+ cells: 0.88 ± 0.01 and 0.87 ± 0.01; aVis+ cells: 0.85 ± 0.02 and 0.85 ± 0.02; aVis− cells: 0.87 ± 0.01 and 0.87 ± 0.01; spatial Information of pVis+ cells: 1.83 ± 0.32 and 2.12 ± 0.36; aVis+ cells: 1.63 ± 0.25 and 1.90 ± 0.39; aVis− cells: 1.78 ± 0.24 and 1.98 ± 0.29, in the uniform and striped mazes respectively; *p* > 0.05 for all comparisons, paired *t* test across conditions, and Kolmogorov-Smirnov test across neurons; *N* = 21 pVis+, 45 aVis+, 31 aVis− FFNs [35]; Fig 4G–4I).

The locations and extent of the place fields were highly correlating in both the uniform and the striped trials. This suggests that the animals considered the two conditions spatially identical despite the different visual percepts (mean Rho = 0.86 ± 0.13; *p* < 0.05 in 51 of 53 neurons, Pearson’s linear correlation of location-resolved FRs in the two conditions, 20 spatially binned FR pairs per neuron, Fig 4J and S5 Fig), and the perceived visual motion caused only a rate remapping [36,37] but not a location remapping of the “place fields.” The spatial locations where the stripedness significantly altered the neuronal activity were determined by comparing the induced firing pattern differences to surrogate datasets (Fig 4B and S3 Fig). Of the aVis+ cells, 124 met the spatially resolved significance criteria (see Materials and methods). The significantly modulated segments of the space-resolved firing patterns uniformly tiled the whole extent of the linear maze; 8.51% ± 0.38% of the 124 aVis+ neurons were modulated at any spatial location (*p* = 0.76, *N* = 2,444, chi-squared goodness-of-fit test against uniform distribution, Fig 4K).

The visual modulation of the firing patterns was stronger when faster visual motion was perceived (i.e., faster running speeds), similarly to the spatiotemporal tuning found in the pVis task (Rho = −0.46, *p* < 0.001, Pearson’s linear correlation of the instantaneous velocity and the visual MIs in *N* = 304 spatial bins from 28 sessions, S6A Fig).

Only a small fraction of the visually responsive neurons modulated their FRs as a function of the instantaneous running speed (*p* < 0.05, Pearson’s linear correlation; number of modulated neurons = 3 of 25 pVis+, 20 of 221 aVis+, 26 of 233 aVis−). The velocity of the animals was identical in the two environments (83.3 ± 18.9 cm/s versus 85.0 ± 12.3 cm/s, *n* = 718 and 649 for uniform and striped conditions, respectively; *p* = 0.15, two-sample Kolmogorov-Smirnov test; S6B Fig).

Although the overall brightness of the “uniform” gray and the black and white “striped” conditions was identical, an alternative explanation of the FR modulation of the aVis+ cells may be that the striped pattern by itself could influence the neuronal activity, either by inducing a different contextual percept or by its local brightness fluctuations. To exclude these alternatives, we performed a set of complementary experiments and analyses.

The rats perceived a relatively stationary representation of the striped and the blank patterns while they stayed at the reward zones (i.e., the last 10 cm of the maze at each end). We compared the firing patterns of the aVis+ cells during these segments to decide if the two contexts induced the FR modulations. The animals spent comparable time in the reward zones (23.11 ± 1.88 s and 26.46 ± 2.14 s, *N* = 1,303 and 1,343 trials for uniform and striped conditions, respectively, *p* = 0.24, two-sample *t* test, S6C Fig), and the mean FRs of the aVis+ cells were almost identical (mean FRs = 9.01 ± 0.89 Hz and 8.99 ± 0.88 Hz for uniform and striped conditions, respectively, *p* = 0.83, *N* = 325 neurons, paired *t* test, S6D Fig). By comparing the neuronal activity on a cell-by-cell and trial-by-trial basis, only 36 of the 325 aVis+ neurons showed significantly different FRs in the two conditions (*p* < 0.05, two-sample *t* test on the per-trial firing patterns for each neurons, S6E Fig).

We also tested if the aVis+ neurons were sensitive to changes of brightness. First, to check their sensitivity for global changes, we exposed the rats to 0.5 Hz blinking light in the pVis environment. S6F Fig shows 2 representative neurons modulated by the global luminance change (*p* < 0.001 in both cases, *N* = 50 trials). Eleven and 2 of the 20 tested pVis+ cells, 5 and 11 of the 111 aVis+ cells, and 7 and 12 of the 124 aVis− neurons, respectively, responded with excitation and inhibition for the luminance increase (*p* < 0.05, paired *t* test, *N* = 50 trials for each neuron; S6F and S6G Fig).

Second, we analyzed FRs in the linear maze stripe cycle by stripe cycle to check if the aVis+ neurons display phasic responses to the gradually changing brightness of each stripe. The aVis+ neurons were not sensitive to the local luminance changes of the black and white stripes. The FRs were nearly uniform at locations with low and high local luminance in the striped condition (*p* = 0.23 and 0.45, *N* = 193 and 358 spikes for the uniform and the striped conditions, respectively; Rayleigh test; S6H Fig shows the stripe phase—resolved firing patterns of a representative example neuron). For all aVis+ neurons, the circular MIs of the FR patterns were similar in the “striped” and the “uniform” trials (*p* = 0.86, two-sample *t* test, mean MI = 13.22% ± 1.50% and 13.61% ± 1.82%, *N* = 93 and 78 aVis+ neurons with nonuniform circular distribution in the uniform and the striped conditions, respectively; S6I Fig).

These observations led us to conclude that the FR change of the aVis+ neurons between the two applied conditions was induced by the perceived visual motion, as all other aspects were kept and were perceived unchanged (i.e., trajectory, reward, global luminance, and other contextual cues).

**Fig 5:**
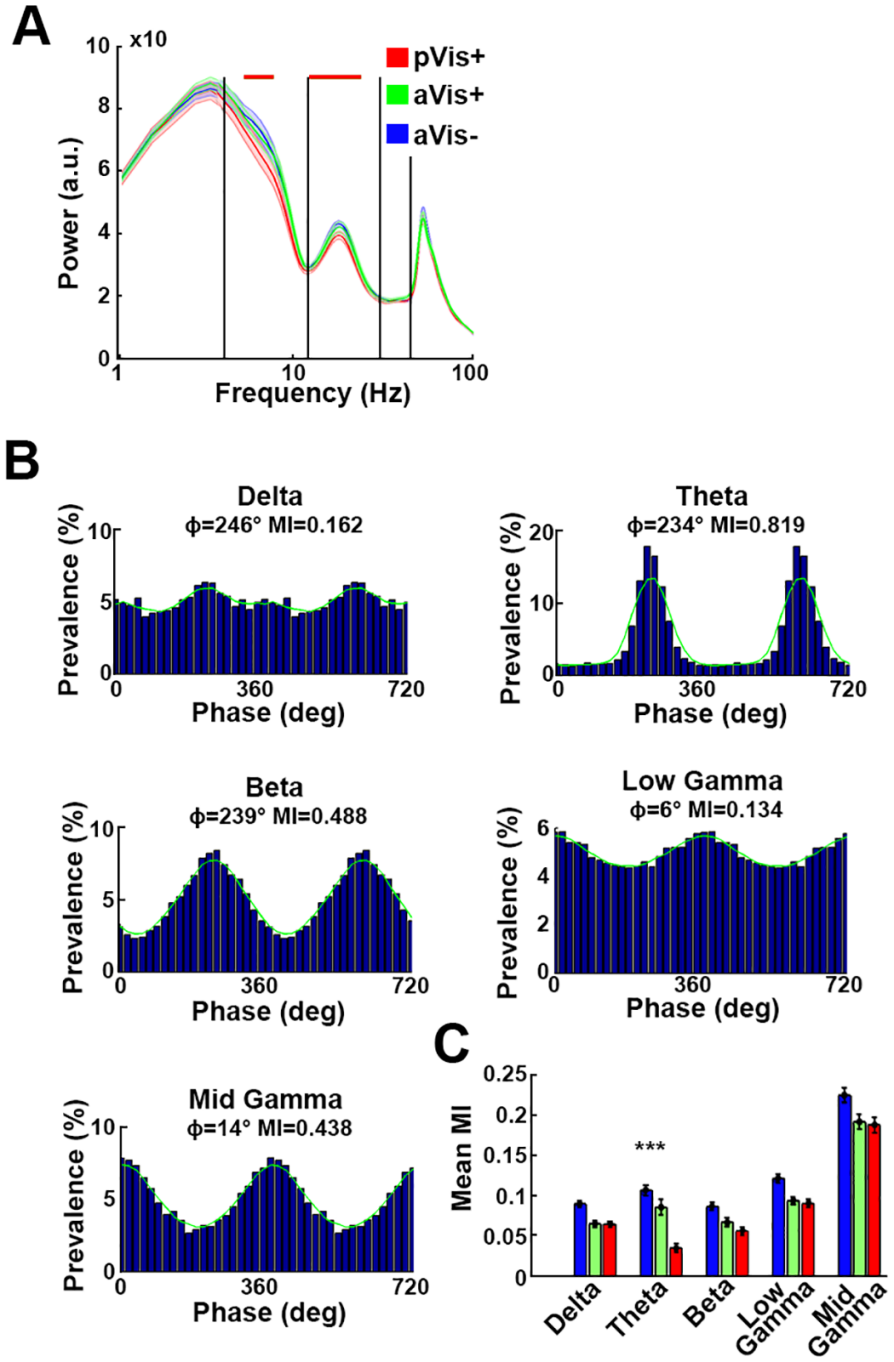
Phase modulation of the recorded CPu neurons by the characteristic frequency bands of the striatal LFP. (A) Mean LFP spectral power at locations of the pVis+ (red), aVis+ (green), and pVis−/aVis− somata (blue). The theta and beta powers were significantly weaker at locations where pVis+ neurons were present (the red horizontal lines show the significant segments). (B) Respective phase coupling of a representative neuron to the delta, theta, beta, low-gamma, and high-gamma bands. The mean preferred phase values and MIs are shown above each plot. Green curves mark the smoothed distributions. (C) Mean MIs of the pVis−/aVis− (blue), aVis+ (green), and pVis+ (red) neurons. The pVis+ neurons were less coupled to the phase of the theta and beta band oscillations. The color codes are the same as on panel A. a.u., arbitrary unit; aVis, active visual; CPu, caudate putamen; LFP, local field potential; MI, modulation index; pVis, passive visual.

The neurons of the CPu were generally phase locked to the characteristic frequency bands of the local field potential (LFP, Fig 5A and 5B, S7 Fig) recorded in the dorsomedial CPu. In general, the pVis+ and aVis+ neurons were more weakly coupled to these oscillations than their pVis−/aVis− peers (mean MIs, delta: 10.84 ± 0.50 versus 7.89 ± 0.50 and 7.85 ± 0.41; theta: 12.89 ± 0.76 versus 10.37 ± 1.16 and 4.29 ± 0.65; beta: 10.48 ± 0.58 versus 8.12 ± 0.65 and 6.80 ± 0.58; low-gamma: 14.64 ± 0.64 versus 11.36 ± 0.53 and 10.93 ± 0.59; high-gamma: 26.96 ± 1.10 versus 23.02 ± 1.12 and 22.57 ± 1.12 for pVis−/aVis− versus aVis+ and pVis+ neurons, respectively). This difference in phase coupling was the most prominent in the theta band (*p* < 0.001, *n* = 145 and 25, Kolmogorov-Smirnov test, Fig 5C).

To check if the anatomically identified cortical regions indeed play a role in shaping the activity of the visual motion—sensitive CPu cells, we lastly set out to investigate their functional connectivity. However, confirming the direct monosynaptic cell-to-cell interactions between the visual cortex and the CPu neurons in intact animals is like looking for a needle in a haystack. Cross-correlation-based analyses are very unreliable with sparsely firing neurons because of the low number of spikes, even if extensive recordings are done for many hours. Optogenetics-based transsynaptic excitation/inhibition is also problematic because of the unknown synaptic transmission efficacy. Moreover, V1 is known to project only to a very thin medial strip of the CPu along its ventricular wall [20]. This is extremely difficult to investigate using electrophysiological approaches in freely moving animals. Because of these constraints, we employed two alternative approaches.

**Fig 6:**
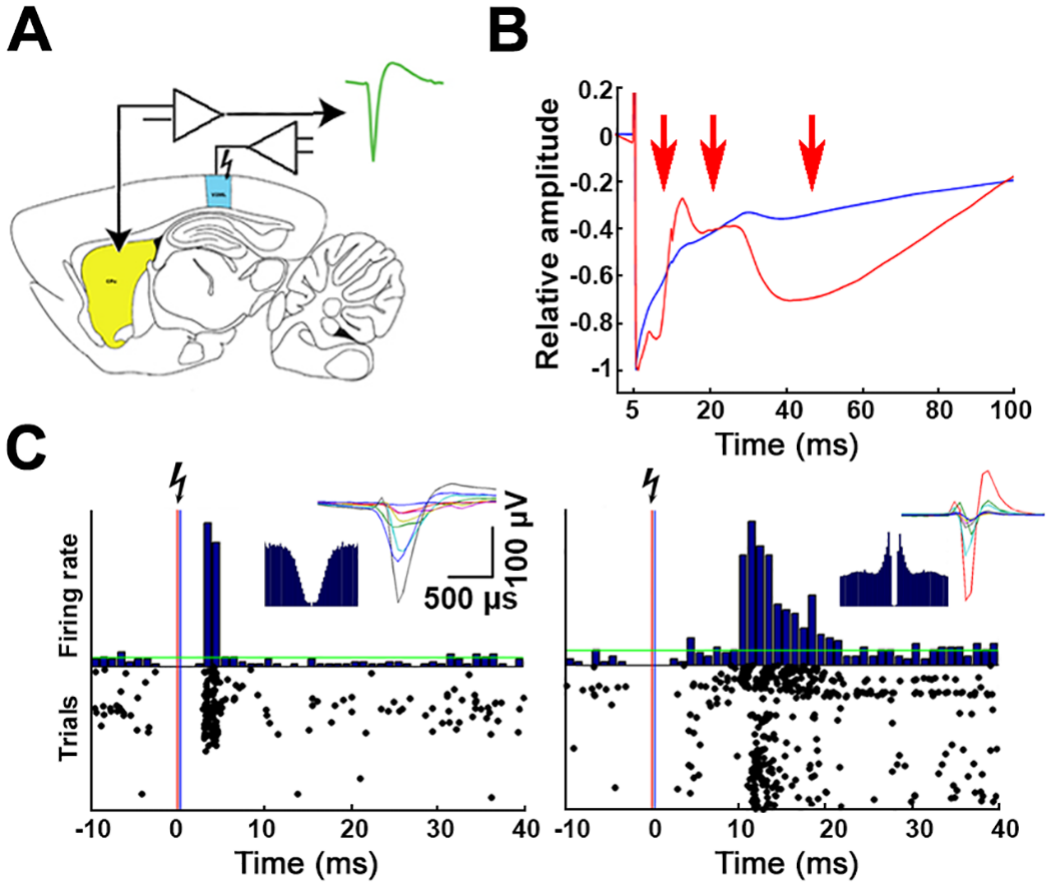
Entrainment of the CPu activity by electrical stimulation of the V2. (A) Schematics of the electrical stimulation. The sites of the cortical stimulation and the striatal recordings are respectively marked by blue and yellow colors. (B) Representative striatal (red) and a control cortical (blue) mean LFP traces as a response to V2 electrical stimulation. Note the 3 remarkable evoked deflections in the striatal LFP (red arrows), which represent the aggregate population activity of the mono-, di-, and polysynaptically activated CPu neurons. (C) Representative peristimulus time histograms and raster plots of a monosynaptically (left panel) and a polysynaptically (right panel) entrained CPu neuron during electrical stimulation of the V2. The insets show the waveforms and autocorrelograms of each example neuron. CPu, caudate putamen; LFP, local field potential; V2, secondary visual cortex.

First, we implanted bipolar-stimulating electrodes into area V2 (AP −4.5 mm; ML 2.8 mm; dorsoventral [DV] 0.5 and 2 mm) of 2 animals. In a subset of sessions, we delivered short electrical pulses (*N* = 21 sessions) to functionally verify the previously confirmed long-range anatomical connection (Fig 6A). The electrically evoked, complex LFP deflections had 3 distinct components whose latency and duration matched with the single unit—evoked responses: a narrow, precisely timed monosynaptic deflection was followed by a wider (presumably disynaptic) one and an even longer polysynaptic waveform. In contrast, at cortical locations above the CPu, only a single monotonic deflection attributed to the volume-conducted stimulus artefact was present (Fig 6B). The brief electrical pulses evoked spiking in 32 out of 289 CPu neurons, either with very short response latency (4–6 ms, representing a monosynaptic input) or through a longer multisynaptic pathway (Fig 6C). The group of neurons responsive to electrical stimulation of area V2 and the group of pVis+ neurons were mainly overlapping; 20 of 25 (80%) pVis+ neurons were also sensitive to electrical stimulation, while the same applied to only 12 of 252 (6%) pVis− cells. Consequently, the intrastriatal distributions of these groups were similar; they were also mainly located at the mediodorsal part of the CPu.

**Fig 7:**
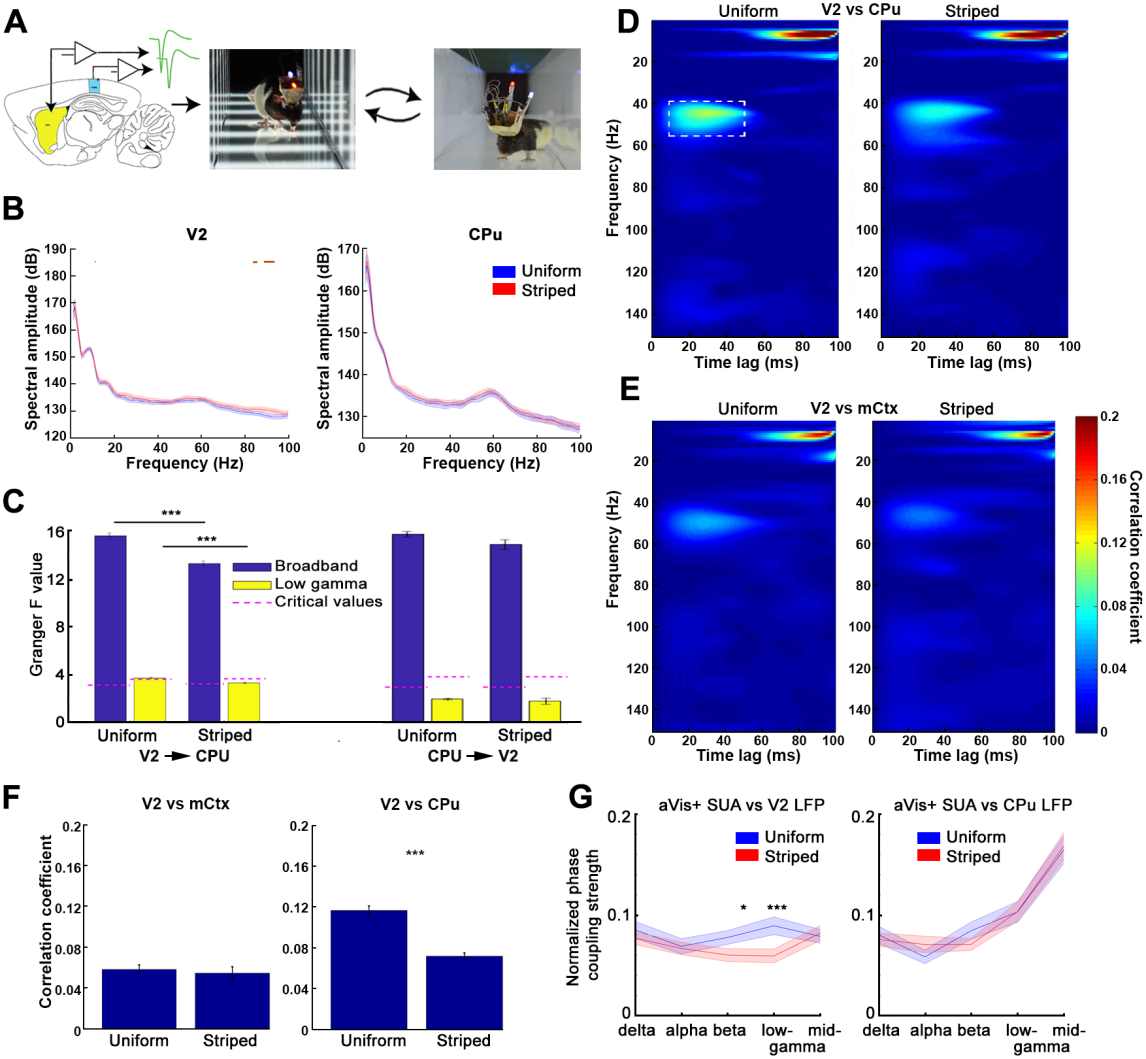
Activity coupling between V2 and the CPu. (A) Experimental schematics showing the simultaneous recording of the LFPs and SUA in both the V2 and the dorsomedial CPu. (B) Mean raw spectra of the V2 (left) and CPu (right) are almost identical when the animals are running in both the uniformly gray (blue lines) and the striped (red) environments (mean power spectra of *N* = 30 and 175 LFP sessions, respectively). The only significant difference was detected in the high-gamma band of the V2 (red horizontal lines). (C) V2 LFP Granger causes striatal LFP oscillations both in the broadband (left panel, blue) and low-gamma filtered signals (left panel, yellow). Note the significant decrease of the causality strength in the striped environment compared to the uniform trials. Right panel denotes the results of the causality analysis in the striato-cortical direction. (D) Average time and frequency—resolved coherence maps across all sessions and animals for trials in the uniform (left) and striped (right) environments. Note the presence of the high-coherence domain between 40 and 50 Hz with a temporal lag of 20–40 ms (V2 leading). (E) Same as in (D) when comparing V2 LFP with the LFP of the mCtx serving as a control. (F) Comparison of the maximal coherence values between 40–55 Hz and 5–50 ms (marked by a dashed white box on (D)). The left and right panels respectively compare the coherences of the mCtx versus V2 and CPu versus V2 during running in both striped and uniform environments. (G) Phase coupling of aVis+ CPu neurons to various frequency bands of the V2 (left) and the striatal (right) LFP during uniform (blue) and striped (red) trials. Note the stripedness-induced decoupling of the aVis+ cells from the V2 beta and low-gamma bands. aVis, active visual cortex; CPu, caudate putamen; LFP, local field potential; mCtx, motor cortex; SUA, single-unit activity; V2, secondary visual cortex.

As a second approach, we implanted an additional 32-channel silicon probe into area V2 in 2 animals. We recorded both single units and LFPs while the animals were exposed to the aVis stimulation protocol in the linear maze (Fig 7A). The frequency spectra of the LFPs recorded at the CPu and at V2 were identical in the striped and uniform trials (except 80–90 Hz in V2, Fig 7B). This suggests that the visual environment does not alter the gross activity of these structures. Still, V2 activity has a role in shaping the activity of the striatum, and its influence is approximately 10%–15% weaker in the striped environment than in the uniform trials. This was shown by the Granger causality between the V2 and CPu broadband and the low-gamma filtered LFPs (V2 → CPU: F_broadband_ = 15.58 ± 0.22 and 13.27 ± 0.2; F_low-gamma_ = 3.63 ± 0.06 and 3.32 ± 0.06; CriticalValue_broadband_ = 3.13 ± 0.008 and 3.25 ± 0.008; CriticalValue_low-gamma_ = 3.63 ± 0.008 and 3.67 ± 0.008; *N* = 3,424 and 4,096 trials in the uniform and the striped conditions, respectively; *p* < 0.001 for both the broadband and low-gamma band comparison of the F-values of all trials; two-sample *t* test; Fig 7C). Regarding the information flow from the CPu to V2, the analysis could not reveal significant causality in the gamma band; however, the broadband causality was maintained. We did not find any difference between the uniform and striped conditions in this case (CPu → V2: F_broadband_ = 15.71 ± 0.24 and 14.85 ± 0.4; F_low-gamma_ = 1.98 ± 0.08 and 1.81 ± 0.25; CriticalValue_broadband_ = 3.01 ± 0.008 and 3.01 ± 0.008; CriticalValue_low-gamma_ = 3.84 ± 0.008 and 3.84 ± 0.008; *N* = 3,424 and 4,096 trials in the uniform and the striped conditions, respectively; *p* = 0.21 and 0.24 for the broadband and low-gamma band comparison of the F-values of all trials, respectively; two-sample *t* test; Fig 7C).

The high level of causality of the broadband signals in the CPu-V2 direction calls for the cautious interpretation of the Granger causality analysis [38]. Traditional correlation and coherence analyses cannot reveal sparse, package-like increases in the synchrony of discrete frequency bands, especially if they are lagging. This is because the asynchronous, uncorrelated majority of the signals hide it. To overcome this bottleneck, we employed a time and frequency—resolved cross-coherence estimation. This is particularly sensitive to lagging, intermittent changes in synchrony, such as “bursty” transmissions (S8 Fig). Using this method, we found that there are transient oscillations in the mid-gamma band that appear coherently in both V2 and the CPu with a constant time lag (V2 leading with approximately 30 ms, Fig 7D). A similar but less coherent delayed pattern was present between V2 and the motor cortex (recorded above the CPu as control, Fig 7E).

In contrast to the V2–motor cortex coherence, which was similar both during running in the uniform and the striped mazes (correlation index = 0.058 ± 0.004 and 0.054 ± 0.006, respectively, for the uniform and striped conditions; *p* = 0.37; *N* = 64 recording-site pairs), the V2 coherence with the CPu became significantly weaker when the striped pattern was present (correlation index = 0.11 ± 0.004 and 0.07 ± 0.003, respectively, for the uniform and striped conditions; *p* < 0.001; *N* = 96 location pairs, Fig 7F). To confirm if the decreased gamma band coherence also has an effect in shaping the striatal unit activity, we tested the LFP phase coupling of the striatal spike trains of the aVis+ neurons. The strength of the phase coupling to local LFP was identical in the uniform and the striped conditions. In contrast (as predicted by the LFP coherence analysis), coupling to the distal V2 LFP was decreased in the beta and low-gamma bands when the animal was running in the striped environment (Fig 7G). These findings suggest the presence of a population-level communication “channel” between V2 and the dorsomedial CPu, which is influenced by the visual environment.

## Discussion

We found a small, distinct group of the dorsomedial striatal neurons that are responsive to both self-motion-independent (passive) and self-motion-congruent (active) visual stimuli (pVis+ neurons). In addition, there is a larger subset of striatal cells that only sense visual motion, when congruent locomotion is simultaneously present (aVis+ neurons). These findings emphasize the importance of investigating visual motion—related sensory processing in freely moving animals instead of anesthetized or head-fixed preparations [9,22,39]. The perceptual capacity represented by these two cell groups may support survival. They allow for the recognition of incongruently moving objects (such as prey). At the same time, due to the self-motion of the observer, they also provide reafferentation about the continuously changing visual environment. Even though the two functional groups we identified in this study shared many electrophysiological properties, our results suggest that pVis+ neurons were overwhelmingly FFNs.

### Identification of striatal cell types from extracellular recordings

Despite their extensive interconnections, the CPu has a very distinct cytoarchitecture from the cortex. The internal circuitry of the CPu is shaped almost exclusively by GABAergic inhibitory neurons, plus a few cholinergic neuromodulator cells. Of these GABAergic cells, 95% are medium spiny neurons forming the functional class of PFNs, while the cholinergic cells are acting as TFNs [40]. The remaining 5% of aspiny GABAergic cells were recently described as a mixture of at least eight morphologically, neurochemically, and electrophysiologically different classes of cells [41–43]. The FFN neurons in this study are most likely PV+, fast-spiking interneurons [27,43,44]. However, the functional classification of the CPu neurons can only putatively and cautiously be matched to the known neurochemical classes. We were able to isolate significantly fewer PFNs and more FFNs (similar to Thorn and Graybiel [45]), as was expected from the results of histological studies with unbiased sampling. PFNs are particularly sparsely firing neurons. Thus, they are the most vulnerable to forced exclusion due to numerical approximation issues or accidentally skipped detection.

### Immediate early gene (IEG) expression in the CPu by visual stimuli

Immunohistochemistry allows for the exact identification of the histochemical cell types in the CPu. However, conclusions about their visual responsivity based on their cFos expression alone should be interpreted with caution. Although the CPu is known to express IEGs in response to cortical stimulation [46], they are not uniformly expressed among different cell types [47]. They can only report increases of FRs reliably [47]. Lastly, it is not possible to distinguish between mono- and polysynaptic activation. We chose to detect cFos over JunB, FosB, or other IEGs for two reasons: first because of its very low constitutive expression in the CPu and second because it is expressed by most cell lines upon stimulation [46]. pVis+ neurons were inhibited by visual motion, which cannot explain the induced cFos expression. We speculate that it was either induced by the blinking light or the stationary grating parts of the stimulation sequence (i.e., more than half of the pVis+ neurons were excited by the change of brightness, and they were also typically excited by the stationary gratings). An alternative explanation is that these neurons were activated through a disinhibition, as the pVis+ neurons were mainly FFNs, but the cFos+ cells were PV−. In this latter case, the cFos+ cells could be mainly medium spiny neurons, which are known to receive inhibitory innervation from the FFNs (putative PV+ fast-spiking interneurons [43]).

### Origin and processing of the visual information in the dorsomedial striatum

Hunnicutt and colleagues [31] reported that neurons of the dorsomedial part of the mouse striatum respond to multiple sensory modalities and that they integrate auditory and visual inputs with emotional and contextual information. The exact drivers of the visual features of the CPu are not clear. We found that aVis+ neurons were sensitive to global (but not local) luminance changes. This finding is in agreement with earlier reports on the large receptive fields of striatal cells spanning almost the whole visual field in cats [29]. These results suggest that the striatum receives a preprocessed “global percept” of the visual environment, instead of a partial pixelated representation.

The cortical discrimination of self-motion-induced visual motion and object motion is generally linked to higher-order visual areas along the dorsal stream of the visual cortex, such as the middle temporal and dorsal medial superior temporal area [39,48,49]. V6 also seems to be able to subtract self-motion-induced dislocations from the visual percept to enhance object motion detectability [48,50]. However, earlier stages of the visual pathway (including V1) can already detect visual motion [51], process features of the motion [52], and even integrate visual motion and locomotion percepts [53]. Upstream stages (such as V2 or V3) inherit these capacities [54] and can serve as a common source for motion-related information to both V5 and V6 pathways [48]. The anterolateral part of area 18a (the lateral extrastriate cortex) of the mouse (which is the homologue of the lateral secondary visual cortex [V2L] and mediolateral secondary visual cortex [V2ML] in the rat [55]) is also linked to the processing of self-motion cues [56]. These V2 neurons prefer lower spatial frequencies compared to V1 and have relatively large receptive fields [54].

Visual motion—sensitive CPu neurons have similar preferences. An earlier report [9] found that caudate nucleus neurons of the cat prefer similar low spatial and high temporal frequencies to what we report here regarding the pVis+ neurons of rats. A direct connection between the visual cortex and dorsocaudal striatum was also confirmed by a few tracing studies in rats and monkeys [20,23,57]. The inhibition of the FFNs by the visual motion in this study suggests an indirect cortical effect through other striatal interneurons (signal inversion). However, this contradicts the direct electrical excitability of the pVis+ neurons. An alternative explanation may be provided by the already negative coding scheme of the corticostriatal afferents from the complex neurons of the V1 [58–60] or, for example, the so-called D cells of the feline extrastriate cortex. These neurons maintain high spontaneous FRs that are decreased when visual motion is perceived [61]. Such decrease in the cortical drive may explain the motion-induced decrease of spiking in the pVis+ neurons, as well as the monosynaptic cortical entrainment that we report here.

Our findings regarding the selective cortical excitability of the pVis+ (but not the aVis+ cells) and their different intrastriatal distribution offer the idea of multiple parallel visual streams as drivers. However, our results only partially support this concept. The more ventral and lateral parts of the dorsomedial CPu (where the aVis+ neurons are also located) did not receive direct V2 input, but the similar response profile in the striped maze indicates that the pVis+ neurons are more likely to be a subtype of the aVis+ group. Nevertheless, as one candidate for the alternative innervation, the lateral-posteromedial nucleus of the thalamus projects to the same striatal and cortical subregions we investigated here [62,63]. This thalamic region may play an important supplemental role in the visual representation of context [64]. However, its effect on the CPu is not yet known. An alternative candidate is the retina—superior colliculus—intralaminar thalamus—CPu pathway [65–67], which converges with tectonigral dopaminergic signals to enhance the detection of unpredictable salient signals [68].

Together, the electrical excitability of pVis+ neurons from the V2 and the corticostriatal gamma band coherence modulated by the visual percept during self-motion suggest a cortical origin for some aspects of the striatal visual information. However, the lack of electrical excitability of aVis+ neurons also predicts the presence of a complementary subcortical input.

The significance of the visual information processing in the striatum has been shown both in visuomotor associations [69,70] and oculomotor functions [8,71]. The exact role of the various striatal aspects of the visual information in shaping the behavior is less clear because studies perturbing selective visual inputs of the striatum are missing. Striatal responses to nonmoving visual stimuli (mainly flashes and other static stimuli) have been unambiguously described in multiple species [7,14,72,73]. However, visual motion is applied mostly in relation to a guided oculomotor or choice task, whose protocols cannot differentiate if CPu neurons respond to the pure visual features of the motion or the abstract, task-associated meaning of the “cue.” The latter explanation is more likely, as responses were reported to enhance over trials of associative learning [74].

Several studies have made attempts to estimate the visual motion—related sensitivity of the striatum [9,10,29]. However, none of them have been performed during natural self-motion. Further experiments are needed to understand the role of striatal representations of the passively observed and self-motion-congruent visual motion percepts in neuronal computations and behavior. Speculatively (as projections of the intralaminar thalamic nuclei converge with visual cortical inputs at the striatum), it is possible that one role of this system is to map the external reflections of self-behavior with salient environmental changes during reinforcement learning [73,75]. In addition to the sensory and motor information, the integration of complex representations such as space, memory, reward, and motivations [76] can serve as a high-utility support to a system like that.

### Multimodal integration in the dorsomedial striatum—Processing nonsensory modalities

Despite the remarkable topographic organization of the striatal afferentations, their functionality is not strictly separated by sharp borders but rather form overlapping gradients [77]. The dorsal part is considered to be an associative zone serving multisensory integration [45]. This segment of the CPu receives a broad repertoire of sensory modalities [7], contextual cues [33], and kinetic and positional information [78], as well as other sources reflecting the internal dynamics of the brain [7,79].

As the animals were running in the linear track, we found a substantial number of neurons possessing place field—like activity [32–34,80,81]. A few of them showed theta phase precession as well [82]. Despite their singular “place fields,” the recorded CPu neurons were relatively weak space coders. Their selectivity and sparsity were approximately half and twice as large, respectively, as what we measured earlier in the case of CA1 place cells [35]. These features may be a projection of the hippocampal activity and not a purely de novo—generated feature, since in an earlier study, they have been found to be more strongly coupled to the hippocampal theta phases than to striatal theta [82]. Nonetheless, besides its possible volume-conducted component, striatal theta is also modulated, for example, by reward signals to some extent [82]. In the case of many neurons, the experimental approach of the current study could not reliably differentiate if the recorded spatial firing pattern was purely related to a coding of the location or if it was also shaped by a reward prediction as the animal was approaching the water ports [83]. The place fields were conserved in both running directions in the maze. Thus, the head direction activity of the striatum [80] might not play a key role in forming these place fields, although we did not investigate the head direction—related patterns in this study.

Besides the spatial information, striatal neurons are also capable of coding various behaviorally relevant environmental contexts [4,40]. To avoid any associations of valence or other behavioral meaning to the “stripedness” of the maze, we designed our aVis stimulation experiment to employ a model-free reinforcement-learning paradigm [84]. The activity of the aVis+ neurons was only partially modulated by the visual stimuli in a similar way to rate remapping [85,86] (i.e., the presence of the striped pattern did not alter the spatial location of the place fields but could modulate the intraplace field FRs [36,37]). This rate modulation was stronger at high temporal frequencies of the perceived visual motion (i.e., at faster running speeds), similarly to the spatiotemporal tuning of the pVis+ neurons in the pVis experiment. Moreover, we found no difference in the FRs of the aVis+ neurons while the animals were not running and were observing the same but steady patterns. The identical running velocities and trajectories in the two conditions ruled out further that the FR difference between the two conditions was induced by the different motor (i.e., locomotion) patterns. Our additional argument against the contextual but not visual motion modulation of the aVis neurons in the linear maze is that the appearance of the motion of identical striped patterns in the pVis environment did not alter the activity of the aVis+ neurons. These observations suggest that the animals did not consider the two conditions as two independent contexts and highlight the integrative nature of the neuronal activity in the striatum.

Traditionally, the hippocampus and the striatum were considered as two independent but complementary memory systems, respectively responsible for episodic and procedural memory [87]. An alternative concept is that the CPu should be better considered as an integrator that has the chance to adjust procedural motor functions based on the internal representations of “past experiences” (i.e., through hippocampal episodic and spatial memory [5]) and the “present circumstances” (i.e., through the contribution of sensory systems). Our finding regarding the coexistence and simultaneous, conjunctive expression of sensory-related and contextual/spatial (e.g., hippocampal) features in the CPu may support this latter concept.

### Network oscillations in the dorsomedial striatum

During their striatal integration, the proper timing of the converging inputs of various modalities is orchestrated by local and global oscillations. The origin and significance of the various LFP oscillations in the CPu are unclear due to the lack of any ordered geometrical alignment of the striatal neurons [88–90]. LFP oscillations can be (1) locally generated by the local reverberating excitatory—inhibitory connections, (2) passively and instantaneously volume-conducted to the site of observation, or (3) evoked locally, driven by rhythmic inputs from distal structures when the local neuronal activity resembles the original oscillation with a synaptic delay. In this third case, the current source density analysis of structures with unordered neurons (e.g., the striatum) will not reveal any characteristic source and sink patterns. Still, as most extracellular recordings in intact animals are referenced to a distal “electroneutral” location (e.g., the cerebellum), even the randomly arranged striatal neurons can give rise to large-amplitude LFP oscillations. This is because they rhythmically sink and release ionic charges from the extracellular space during their synchronized discharges [91,92]. In the striatum, all three ways most likely contribute to the generation of LFP oscillations.

Although theta phase coupling is present in a good number of striatal neurons, in vitro studies have not revealed any cellular properties that may support the local generation of the theta rhythms [93]. Since phase precession is more constrained to hippocampal theta than to the local rhythm [82], it is likely that the CPu only resembles the theta-modulated activity from the hippocampus or connected structures (e.g., prefrontal cortex [94,95]). Similarly, delta oscillations are imposed by corticostriatal neurons driving striatal interneurons and medium spiny neurons [44,96]. However, beta band oscillation can be generated by the CPu on its own through the interplay between PV+ fast-spiking interneurons and medium spiny neurons under the control of dopamine neuromodulation [97].

The picture is not so clear in the case of gamma oscillations. Van der Meer and colleagues [77] found that at least part of the ventral striatal gamma activity is locally generated. However, the CPu network lacks the necessary inhibitory—excitatory loop that could generate these rhythms alone [98,99]. Along these lines, it is likely that rhythmicity is generated elsewhere (e.g., the hippocampal formation, the piriform, or the visual cortex) and that the striatal neurons only resonate with these inputs [88,89]. However, the synchronized, sudden switches between 50 and 80 Hz gamma rhythms along the whole extent of the ventral striatum also suggest the presence of some coherent internal mechanisms [83].

The distal and local generation of the respective lower- and higher-frequency oscillations is reinforced by the observation that the activation of TFNs selectively increases the power of both the beta and gamma bands but not the lower-frequency bands [97]. We strongly hypothesize that the LFP oscillations of the dorsal striatum are at least partially generated by the coordinated discharges of the striatal neurons that are governed by their distal inputs (including the visual cortex). This idea is supported by the lagging gamma band coherence of the striatal and V2 LFP oscillations, as well as the differential phase locking of the recorded neurons to local and distal gamma oscillations.

### The possible role of the corticostriatal LFP oscillations

We found that the neurons of V2 express oscillations in the low- and mid-gamma band. Such patterns also appear in the CPu with approximately 20–30 ms delay (called gamma-50 by van der Meer and Redish [83]). We interpret this delay as a sign of information transfer between the visual cortex and the striatum. This delayed synchrony is not present at other frequency bands (except for the theta band), and it quickly decays, even if the signals are further shifted with an integer number of cycles. This suggests that the detected events are discrete packages of a few gamma cycles but not a persistent gamma oscillation (see S8 Fig for interpretation). The lag of these gamma epochs is in agreement with the latency of the striatal excitatory postsynaptic potentials (EPSPs) compared to the V1-evoked potentials after visual stimulation, as reported by Reig and Silberberg [7]. We assume that these striatal gamma events are likely to be generated by the evoked gamma frequency bursts of PV+ fast-spiking interneurons [100], since similar PV+ interneurons are responsible for gamma oscillations in the hippocampus and cortex [101]. This hypothesis is compatible with our finding that the majority of pVis+ neurons are FFNs, underpinning that their modulation through sensory inputs may have an effect on the generated gamma events. We found that visual stimulation decreases the FR of the FFNs and presumably their cortical afferents as well. Thus, we assume that this decreased cortical “constrain” is the cause of the weaker gamma coherence in the striped maze.

Coherent LFP oscillations serve as “semaphores” for interactions between many circuits of the brain and are efficient filters for information transfer [102]. The detected oscillatory delay of the mid-gamma band matches the delay of the monosynaptic responses recorded in our electrical stimulation experiment. It is therefore possible that its purpose is to offer highly excitable time slots when the CPu neurons become more sensitive to cortical information. It is doubtful whether these gamma patterns are related only to the visual cortical interactions or that multiple inputs are converging to the CPu during these short temporal windows, given that the importance of the striatal gamma patterns has already been shown in signal response—association tasks [103]. On the other hand, the weak theta phase coupling of the pVis+ neurons reflects their relatively steady responsivity. This is a prerequisite for the reliable quick recognition of moving objects at any moment. Further experiments are needed to gain evidence to verify these hypotheses.

Differential innervation conclusively distinguishes pVis+ cells from the larger pool of perceptors detecting visual dislocations induced by self-motion (i.e., aVis+ neurons) and establishes them as a separate functional group. We propose that the aVis+ neurons may provide robust feedback on the dislocation of the gait caused by the movements of the animal. In addition, successive comparison of the activity of aVis+ and pVis+ neurons can extract the presence of independently moving third-party objects such as prey or predators in a nonstationary (but otherwise neutral) visual environment. This process may be important in detecting self-independent moving objects and for selecting the appropriate action to challenge them. Moreover, the aVis+ and pVis+ neurons also integrate spatial (and possibly contextual and kinetic) modalities. They are modulated by visual motion co-occurring with self-motion whose features may have a utility in sensorimotor integration while performing the chosen action. Further experiments may confirm these hypotheses by revealing the origin of the visual information that drives the aVis+ neurons and testing the behavioral consequence of the selective manipulation of these two pathways. Optogenetic silencing of the specific axon terminals in the dorsomedial CPu may be a good approach to achieve this in the future.

## Materials and methods

### Ethics statement

All experiments were approved by the Ethical Committee for Animal Research at the Albert Szent-Györgyi Medical and Pharmaceutical Center of the University of Szeged (No. XIV/218/2016 and XIV/471/2012). Thirty-two male rats (Long-Evans, 3–12 mo old) were used in this study.

### Awake electrophysiological experiments

#### Surgery and recordings

Electrode fabrication and implantation surgery were described in details earlier [26,104]. Briefly, animals were anesthetized with isoflurane, and 1 or several craniotomies were performed under stereotaxic guidance. Sixty-four channel silicon probes (NeuroNexus, Ann-Arbor) were implanted in the dorsomedial striatum. In 2 animals, an additional 32 channel silicon probe was implanted in the visual cortex of the same hemisphere. In 2 other animals, a bipolar stimulation electrode made of two 50 μm thick parylene-insulated tungsten wires was implanted in the visual cortex (AP 4.5 mm posterior from the bregma; ML 2.8 mm; DV 0.5 and 2 mm, 1,500 μm distance between the tips of the wires, 500 μm uninsulated part at each tip). Silicon probes were mounted on custom-made micro-drives to allow their precise vertical movement after implantation. The probes were inserted above the target region, and the micro-drives were attached to the skull with dental cement. The craniotomies were sealed with sterile silicone. Two stainless steel screws were drilled bilaterally over the cerebellum, serving as ground and reference for the recordings. Several additional screws were driven into the skull and covered with dental cement to strengthen the implant. Finally, a copper mesh was attached to the skull with dental cement and connected to the ground screw to act as a Faraday cage, preventing the contamination of the recordings by environmental electric noise. After postsurgery recovery, the probes were moved gradually in 75 to 150 μm steps per day until the desired position was reached [104]. The CPu was identified physiologically by increased unit activity. The exact electrode locations were identified post hoc from histological sections.

#### Data acquisition

The operated animals were housed in individual cages and recorded during different navigational tasks. One animal was also recorded during sleep in his home cage. Neuronal recordings were performed daily by connecting the probes to a signal multiplexing headstage attached to a thin and light cable pending from the room ceiling on a trolley system that allowed free movement of the animal. The spatial position of the rats during behavioral sessions was monitored using video tracking of 2 LEDs fixed to the headstage at 30 frames per second, with approximately 3 mm resolution. The wide-band signal (0.2–10 kHz) acquired at 20K Sample/s (KJE-1001, Amplipex Ltd, Hungary) was low-pass filtered and downsampled to 1,250 Hz for LFP analysis and high-pass filtered (>0.5 kHz) for spike detection.

#### Electrical and passive (non-self-motion-induced or self-independent) visual stimulations

A home cage—like visual stimulating environment was created to test the neuronal responses to visual stimuli while the animal was performing nonguided behavior. A 60 × 60 cm rectangular wooden platform was surrounded by four 24” LED monitors (24M45H-B, LG, Seoul, South Korea) serving as approximately 30 cm tall walls, and the rats were placed in this stimulation box. One stimulus trial consisted of an initial 1 s uniform gray screen, 1 s of full-screen stationary grating with sinusoidally modulated luminance in a given orientation (8 orientations in 45° steps) and with a given spatial density (170, 85, or 42.5 mm/cycle), and finally 1 s of moving grating, during which the aforementioned grating was sliding orthogonal to its orientation with a given temporal velocity (1,200, 600, or 300 mm/s). The overall brightness of the three conditions was adjusted to be isoluminant (i.e., gray screen versus black-to-white-to-black gratings). The spatial densities and temporal velocities of the gratings were chosen to match the spectral preference of the caudate neurons reported earlier, when viewed from the center of the cage, and were combined in a way that gave a constant sliding speed of approximately 7 cycles/s [9]. The frame rate of the monitors was set to 60 Hz; thus, the grating was sled with approximately one-ninth of a cycle in each consecutive frame, which gave the percept of a smooth motion. A pseudorandom sequence of visual stimulus trials was generated by random combinations of direction/orientation (8) and spatial/temporal properties (3), interleaved with 3 s long trials of blinking full-screen light stimulation at 0.5 Hz (i.e., a full-screen 1 s black–1 s white–1 s black sequence). The final sequence of two thousand five hundred 3 s long trials was played on all 4 monitors simultaneously. Each parameter combination was repeated 100 times. The displayed frames were always identical on the 4 monitors, and the room was otherwise completely dark. The visual stimulation was synchronized with the neuronal data by simultaneously recording a digital code displayed at the top-left corner of the screens by a photodetector. This 1 × 1 cm large visual code was not visible to the animals, as these parts of the monitors were covered. Animals’ behavior was monitored using a web camera, and trials in which they were sleeping instead of random exploration or quite wakefulness were excluded. The same stimulation sequence was used for the initial screening of intrastriatal cFos distribution. To test the sensitivity of the neurons to luminance change, only the trials of the full-screen flash patterns were analyzed. All stimulation protocol was performed during the dark cycle of the animals.

In animals with stimulating electrodes in the visual cortex, we also tested single-unit and evoked-potential responses in the CPu for electrical stimulation. Bipolar electrical pulses (300 μs long 500 μA pulses repeated every 300 ms for 5 min during each session) were delivered, and CPu recordings were performed while the animals were kept in the same stimulating cage as above, with the monitors displaying the uniform gray screens.

#### Active (self-motion-induced) visual stimulation in the linear maze with dynamically updatable visual environment

In order to investigate the responses of CPu neurons to visual motion induced by the self-motion of the animals, we constructed a linear maze in which the visual environment could be randomly and instantaneously swapped between uniform grayness and sinusoidally modulated gratings while the animal was running for water rewards in the maze. The overall brightness of the two conditions was isoluminant. The visual scene was projected onto the walls and floor of the maze using a video projector (MW663, BenQ, Taipei, Taiwan) in an otherwise dark room. To prevent the animals from casting a shadow and blocking the projection on parts of the environment, which is a bottleneck of projecting from above, the maze was constructed of translucent acrylic glass, and the scene was projected on these walls from outside, using a dual mirror system (see Fig 4A). The rats were trained to run back and forth in this maze to collect small water rewards. These droplets were automatically delivered at water ports located at the two ends of the maze when the animal passed through the infrared gates at the opposite side. These gates were located 10 cm away from the water ports. After each run, the projected scene was randomly swapped between a uniform gray image and a stationary grating. To match the preferred spatial density of the passive stimulation, 16 grating cycles were projected along the full length (230 cm) of the maze. As the rats were running at about 85 cm/s speed, the temporal velocity of the apparent visual motion (approximately 6 cycles/s) was comparable to that used in the passive stimulation environment.

#### Postmortem verification of the electrode locations

Following the termination of the experiments, animals were deeply anesthetized. All animals were transcardially perfused first with 0.9% saline solution followed by 4% paraformaldehyde (PFA) solution. Brains were sectioned in 60 μm thick slices (VT1000S Vibratome, Leica, Wetzlar, Germany) in the coronal plane and stained using 4′,6-Diamidino-2-phenylindole dihydrochloride (DAPI; D8417, Sigma-Aldrich). Locations of the electrode tracks were identified using epifluorescent microscopy (AxioImager, Carl Zeiss, Oberkochen, Germany).

### Anatomical tract—tracing experiments

Long-Evans rats (260–580 g) of both sexes were anesthetized with 1%–3% isoflurane and then mounted on a stereotaxic apparatus. Atropine (0.1 mg/kg, s.c.) was administered immediately after the anesthesia induction. Stages of anesthesia were maintained by confirming the lack of vibrissal movements and nociceptive reflex. The rectal temperature was maintained at 35–37 °C with a DC temperature controller (TMP-5b; Supertech, Pécs, Hungary). A small incision on the skin was then made for craniotomy after s.c. lidocaine injections.

#### Anterograde tract tracing of corticostriatal neurons

A craniotomy above the right V2 was made 4.0–5.0 mm posterior from the bregma, 2.0–3.0 mm lateral of the midline [105]. A glass capillary (tip 10–20 μm) filled with 10% BDA (D1956; Thermo Fisher Scientific, Waltham, MA) in 0.1 M phosphate-buffered saline (PBS) was installed with an auto-nanoliter injector (Nanoject II; Drummond Scientific, Broomall, PA) and then inserted into the V2 according to the following 4 stereotaxic coordinates: (in mm) AP −4.50, ML 2.25; AP −4.50, ML 2.75; AP −5.00, ML 2.25; AP −5.5, ML 2.25. For each coordinate, 0.3 μl of the BDA solution was ejected at 0.7, 0.85, and 1 mm below the pia at a rate of 0.09 nl/s. After ejections, the capillary tip was held in the position for at least 5 min and then gently retracted. The craniotomy was covered with sterile bone wax, and the skin wound was sutured. After 1 wk of survival, the rats were deeply anesthetized and perfused, and the brains were processed (see section Tissue processing and immunohistochemistry).

#### Retrograde labeling of striatal afferents

Four Long-Evans rats were anesthetized and then mounted on a stereotaxic apparatus as described in section Anatomical tract—tracing experiments. A craniotomy above the striatum (CPu) was made. A glass capillary filled with 4% fluorogold (Fluoro-Gold; Fluorochrome, Denver, CO) in 0.9% saline was installed with an auto-nanoliter injector (Nanoject II) and then inserted into the CPu according to the following coordinates: (in mm) AP −1.25–1.75, ML 3.6–4.0, and DV 3–4 from the bregma, midline, and pia, respectively. For each coordinate, 0.15–0.30 μl of the solution was gently ejected. After 1 wk of survival, the rats were transcardially perfused and fixed; 60 μm thick coronal brain sections were prepared and processed as described in section Tissue processing and immunohistochemistry.

### Tracing experiments in combination with visual stimulation

Nine rats underwent the BDA injection of the anterograde tracing as described in section Anatomical tract—tracing experiments. After 1 wk of survival, the rats were reared in the cage for pVis stimulation (see above) in complete darkness overnight, and then 5 of them were stimulated by the same pVis stimulation used in the freely moving electrophysiological experiments. The 4 other animals were left in the darkness for the same duration (see Fig 2A). After that, the rats were immediately anesthetized and perfused, and their brains were processed for cFos and BDA immunohistochemistry as described in section Tissue processing and immunohistochemistry. In 1 animal, we also tested the identity of cFos+ cells with immunohistochemistry against PV.

### Tissue processing and immunohistochemistry

Following the termination of the experiments, animals were deeply anesthetized. All animals were transcardially perfused first with 0.9% saline solution followed by 4% PFA solution. Brains were sectioned in 50–70 μm thick coronal slices (VT1000S Vibratome). The following staining procedures were performed at room temperature.

#### Immunohistochemistry for retrograde tracing

Sections were incubated successively with 10% NGS in PBS containing 0.3% Triton X-100 (PBS-X) for 30 min, 1:2,000 diluted anti-fluorogold polyclonal antibody (AB153-I; EMD Millipore, Billerica MA) in PBS-XG for 48 h, and 4 μg/ml Alexa Fluor 633-conjugated goat anti-rabbit IgG (A-21071; Thermo Fisher Scientific) in PBS-XG for 2 h. Sections were counterstained with a fluoro-Nissl solution for 60 min, washed with PBS, mounted, and coverslipped.

#### Immunohistochemistry for the anterograde tracing experiments combined with visual stimulation

Animals were transcardially perfused with physiological saline followed by 4% PFA and 0.2% picric acid in 0.1 M phosphate buffer (PB; pH 7.2–7.3). After removal, brains were postfixed overnight, embedded in 4% agarose, coronally sectioned at 50 μm thick using a vibrating blade microtome, and harvested in PBS. All incubations were followed by washing with PBS-X. Sections were incubated successively with 10% normal goat serum (NGS) in PBS-X for 30 min, 1 μg/ml rabbit anti-cFos polyclonal antibody (ABE457; EMD Millipore; Kim et al., 2014) in PBS-X containing 1% NGS and 0.02% sodium azide (PBS-XG) overnight, and a mixture solution containing 4 μg/ml Alexa Fluor 488-conjugated goat anti-rabbit IgG (A-11034; Thermo Fisher Scientific) and 2 μg/ml Alexa Fluor 555-conjugated streptavidin (S-21381; Thermo Fisher Scientific) in PBS-XG for 2 h. Sections were then mounted on glass slides, counterstained with a fluoro-Nissl solution (N-21479; Thermo Fisher Scientific) for 40 min, washed with PBS, and finally coverslipped with 50% glycerol and 2.5% triethylene diamine in PBS [106].

#### cFos and PV double immunohistochemistry

Sections were incubated successively with 3% bovine serum albumin (BSA) in PBS-X for 30 min, a mixture of first antibodies (rabbit anti-cFos at 1:000: ABE457; EMD Millipore and mouse anti-PV at 1:3,000: PV235; Swant, Marly, Switzerland) in PBS-X containing 1% BSA (PBS-XBSA) overnight, and a mixture solution containing 4 μg/ml Alexa Fluor 488-conjugated goat anti-rabbit IgG and 4 μg/ml Alexa Fluor 594-donkey anti-mouse IgG (A-21203; Thermo Fisher Scientific) in PBS-XBSA for 2 h. Sections were then mounted, counterstained, and coverslipped.

#### Confocal microscopy

Images of fluorescently stained sections were captured using a laser scanning confocal microscopy (LSM880; Carl Zeiss). Tile images of 5 × 5 and 10 × 10 for the visual cortex and the striatum were acquired, respectively. Each tile of 10 μm optical thicknesses was taken using a Plan-Apochromat 20×/0.8 M27 objective lens (Carl Zeiss), 1.03 μs pixel time, and 4 times frame average at 512 × 512 resolution. The power of the lasers was 0.4–0.7 mW, which did not induce obvious fading.

### Data analysis

#### Statistics and data availability

All data analyses were performed in MATLAB (Mathworks, Natick, MA), unless otherwise noted. No specific analysis to estimate minimal population sample was used, but the number of animals, trials, and recorded cells was larger or similar to those employed in previous works, e.g., [4,83,107]. No randomization or blinding was employed. For statistical testing, Student *t* test, Wilcoxon rank-sum test, or Kolmogorov-Smirnov tests were used, depending on the distribution of the data and the sample size (two-tailed). To compensate the cumulative error due to multiple comparisons, Bonferroni correction was employed. One, two, or three asterisks on the figures denote significance levels <0.05, <0.01, and <0.005, respectively. MIs were always calculated as the ratio of the difference and the sum of two measures. The data that support the findings of this study are available from the corresponding author upon reasonable request, and the numerical values used to generate figure plots can be found in S1 Data.

### Automated processing of the microscopic images

Separate color channels of the raw data were exported as 8-bit tiff files. For cFos and PV double immunostaining, cFos-positive, PV-positive, and cFos- and PV-double-positive neurons were manually counted using the Cell counter plugin of ImageJ software (NIH, Bethesda, MD). For the estimation of BDA-labeled axon density and cFos density, the following automated procedure has been employed (see also S1 Fig): For each slice, a binary mask layer was created in Photoshop (Adobe systems, San Jose, CA) to void the extra-CPu regions (non-CPu mask) and the obvious artefacts of the BDA channel (intra-CPu mask, e.g. blood vessels). The BDA, the cFos, and the mask channels were saved as separate grayscale image files. The cFos image was preprocessed in ImageJ (v1.50) using the Image-based Tool for Counting Nuclei plugin (ITCN, Kuo and Byun, Center for Bio-image Informatics at UC Santa Barbara, https://imagej.nih.gov/ij/plugins/itcn.html) after image inversion. ITCN (settings: width: 14 px, minimum distance 7 px, threshold 1, detect dark peaks) marked the cFos+ somata as red pixels on the grayscale image. All three image files were loaded in MATLAB and divided into 512 × 512 pixel subregions (10 × 10 pieces). cFos density was measured at the corresponding image as the number of red (somata) pixels in each subregion that were not masked in the mask map, normalized by the nonmasked area. BDA image was thresholded using the mean +4 SD luminosity level of the overall nonmasked image. Pixels brighter than this threshold were considered as BDA-labeled axons. BDA density was calculated the same way as for the cFos density. Intra-CPu masks were covering less than 2% of the total CPu area at all slices and were uniformly distributed both along the DV and ML axes and thus did not bias the measured densities. Data of subregions smaller than 2,600 pixels (due to the non-CPu mask) were discarded to avoid numeric approximation errors. ML and DV extent of each CPU slice was normalized when analyzing multiple slices together.

#### Spike sorting and unit classification

Neuronal spikes were detected from the digitally high-pass-filtered raw signal (0.5–5 kHz) by a threshold crossing—based algorithm (Spikedetekt2; https://github.com/klusta-team/spikedetekt2). Detected spikes were automatically sorted using the masked EM algorithm for Gaussian mixtures implemented in KlustaKwik2 [24] (https://github.com/klusta-team/klustakwik/), followed by manual adjustment of the clusters using KlustaViewa software [25] (https://github.com/klusta-team/klustaviewa/) to obtain well-isolated single units. Multiunit or noise clusters were discarded for the analysis. Cluster isolation quality was estimated by calculating the isolation distance and interspike interval (ISI) index for each cluster [26]; poor quality clusters were discarded. Striatal neurons were classified as reported by Schmitzer-Torbert and Redish [107], assisted by monosynaptic excitatory and inhibitory interactions between simultaneously recorded, well-isolated units [35]. Briefly, 5 parameters were calculated: spike width and spike trough-to-peak time [26], FR, proportion of interspike intervals exceeding 2 s (ISI2s), and postspike suppression time (PSST, [107]). Neurons with more than 2% ISI2s, broad spikes, and low FR and PSST were classified as TFNs; those with less than 2% ISI2s, narrow spikes, high FR, and low PSST were considered as FFNs; and finally, those with less than 2% ISI2s, low FR, and high PSST were classified as TFNs. Spike waveforms were extracted ([−1, +1] ms around the threshold crossings) from the raw unfiltered recordings, and their linear trends were removed.

#### Effect of the pVis stimulation on the FRs

Peristimulus time histograms were constructed from the action potential time series, triggered by the visual stimulation sequence. FRs were calculated for the periods during uniformly gray, stationary grating, and moving grating stimulation separately, for each stimulus condition. FR MIs were calculated for each condition for uniform versus stationary and uniform versus moving grating segments as MI_stationer_ = (FR_stationer_ − FR_uniform_) / (FR_stationer_ + FR_uniform_), and MI_motion_ = (FR_moving_ − FR_uniform_) / (FR_moving_ + FR_uniform_). The same approach was used to compare the neuronal activity in dark and brightness during the luminance test. The effect of the blinking light was judged by the trial-by-trial comparison of the FRs during the black and the white periods of the stimulation.

#### Spiking activity in the linear maze

Coordinates of the detected LEDs were linearized by projecting them onto the axis of the linear maze, smoothed using a 10 frames wide Gaussian kernel, and rounded to 1% precision. Successful running trials were considered as periods in the recordings when the animals were traversing from 10% to 90% of the maze or vice versa. Automated trial extraction was performed by detecting 50% crossings in the time series of the linearized positions, and each of the crossing detections were tracked backward and forward in time until the velocity dropped below 5 cm/s. Trials in which either of these automatically detected start and stop positions were between 10% and 90% (i.e., the rat stopped in the middle of the maze) were discarded. Running directions were arbitrarily named “forward” and “backward” runs. Spatially resolved FR histograms and raster plots were compensated by the occupancy frequency of each spatial bin (20 bins) [30]. To determine the responsivity of the neurons in the aVis task, the mean compensated FRs within these spatial bins were compared between the uniform and striped trials using a paired *t* test. MIs were calculated to express the strength of the overall effect of perceived visual motion by comparing the FRs during “striped” and “uniform” trials as MI = (FR_striped_ − FR_uniform_) / (FR_striped_ + FR_uniform_).

The spatial extent of the significant modulation of the neuronal FRs by the visual motion was tested by a label-shuffling approach described in [30]. Briefly, the uniform/striped labels of the trials in each session were shuffled, and the difference of spatially resolved firing patterns during the shuffled “uniform” and the shuffled “striped” trials were calculated. This was repeated 1,000 times to create a surrogate dataset of shuffled differences. Local confidence boundaries of the shuffled datasets were determined at the 2.5%–2.5% tails of the distributions of the shuffled difference values at each spatial bin (“local significance levels”). To compensate the cumulative error of multiple comparison, a “global significance level” was subsequently determined in an iterative way by increasing the local significance levels until only 5% of the shuffled surrogate dataset broke the global threshold. This global significance level was used then to determine the significance of difference of the real, unshuffled firing patterns, smoothed by a 5 bin wide (around 15 cm) moving average.

To correlate the modulation of the FRs with the temporal frequency of the perceived visual motion (i.e., the running speed), the instantaneous running speeds were binned and averaged in 10-cm-wide spatial bins, and the median of the corresponding MIs of the simultaneously recorded aVis+ neurons were calculated in each bin. Sessions with fewer than 3 aVis+ neurons and bins with nonsense MIs (NaNs and Infs due to undersampling errors) were excluded. Because of the low number of spikes in the spatial bins, we calculated the median of the bin-wise MIs of the aVis+ cells within each trial. Extreme (nonsense) median MIs were further excluded by taking the median of the binned MIs with similar corresponding velocities across trials of a given session. These cleaned MIs were correlated then with the corresponding instantaneous running speeds.

Space-resolved neuronal activity patterns were also analyzed with respect to the local luminance at the instantaneous positions of the rats: each location was assigned with the local luminance value of the “striped” pattern (expressed as the phase of the sinusoidally modulated luminance grating). Circular histogram of the “stripe phase”–resolved activity pattern was generated by averaging the activity patterns across all 14 cycles and all trials of the given condition. For the “uniform” condition with nonmodulated gray pattern, the same cycle phase values were used in the analysis as determined in the “striped” condition. Activity patterns were tested for circular uniformity using the Rayleigh test, and only those neurons were included in the population analysis that showed a significant deviation from the von Mises distribution either in the “striped” or in the “uniform” condition. This deviation was quantified as the MI of circular nonuniformity (MI = [FR_peak_ − FR_peak-180°_] / [FR_peak_ + FR_peak-180°_]).

#### LFP spectral analysis and spike—LFP coupling

Power spectrum and time-resolved spectral analysis were performed on the low-pass-filtered and downsampled (1,250 S/s) LFP signals using multitaper spectral analysis. Spectra were whitened by multiplying with the corresponding frequencies and expressed as decibels (dB). For all local spike—LFP coupling analyses, the median of the local LFP signals recorded on the same shank has been used. For phase coupling of the CPu neurons to V2 LFP, the median LFP of all V2 recording sites was used instead. Subjectively noisy LFP channels due to recording sites with too-high impedance were discarded. LFP signals were filtered in 5 frequency bands (delta: [1–4 Hz], theta: [4–12 Hz], beta: [12–30 Hz], low-gamma: [30–45 Hz], mid-gamma: [45–80 Hz]) with an eighth-order zero-phase-lag Butterworth filter, and phase values were obtained using Hilbert transform. No 50 Hz notch filtering was used in the data acquisition or during the data analysis. LFP phase histograms were constructed by taking the corresponding instantaneous LFP phase values corresponding to the isolated single-unit spikes, and their deviation from the circular uniform distribution was tested using the Rayleigh test. The bin size of the histograms was 18° and was smoothed by calculating a 5 bin wide moving average of the histograms. The phase values corresponding to the peaks and troughs of the smoothed distributions were taken as preferred and unpreferred phases, respectively. Phase MIs were calculated from smoothed occurrence frequencies of spikes at the preferred and unpreferred phases to account for possible asymmetric distributions (MI = [FR_max_ − FR_min_] / [FR_max_ + FR_min_]).

#### Spatial location—related activity in the linear maze

Rate map, spatial information, selectivity, and sparsity [35] were calculated for each direction separately. A Gaussian kernel (SD = 5 cm) was applied to both raw maps of spike and occupancy, and a smoothed rate map was constructed by dividing the smoothed spike map by the smoothed occupancy map. A place field was defined as a continuous region of at least 15 cm where the FR was above 10% of the peak rate in the maze, the peak FR was >2 Hz, and the spatial coherence was >0.7. Place fields with fewer than 50 spikes, those that included the turning position of the track, and truncated or overlapping fields were discarded [35,104]. Place fields in both running directions in the linear track were treated independently. Stability of place fields across conditions was tested by calculating the spatial correlation of the binned mean firing patterns in the striped and uniform trials.

#### Cross-region LFP activity analysis

Median LFP signals were calculated for each recording shank (8 recording sites per shank, 8 CPu shanks, and 4 cortical shanks). Granger causality was estimated for each V2 and CPu signal pairs. Only time lags in the range of [2, 40] ms were considered, and the lag with the highest causality measure was displayed. F-statistics were compared with paired *t* test between the uniform and striped conditions. Time and frequency—resolved cross-correlations were calculated for each shank pairs (32 in total) the following way: Both signals were iteratively filtered between 1 and 100 Hz in 1 Hz steps, with an eighth-order, zero-phase-lag passband filters, and their cross-correlations were calculated for [−100, +100] ms lags, in 2 ms steps. The magnitudes of the cross-correlograms were taken as the absolute values of its Hilbert transforms for each frequency. To remove the additive distortion caused by the symmetric correlation of the synchronous events (e.g., common noise), the minima of each symmetric time bin pairs have been subtracted from the same bins. The principle of this approach is demonstrated by analyzing 2 artificial LFP signals and explained on S8 Fig.

## Supporting information

S1 Data (XLSX)

S1 Fig Steps of the automated detection of cFos+ cell and BDA-labeled axon density and cortical cFos expression. (A) Original tile-scan image of the BDA-labeled axons on a representative example slice (left). Middle panel shows the intensity-thresholded image, while right panel denotes the calculated binned axon density. (B) Original tile-scan of the cFos+ neuronal somata on the same slice (left) and its inverted version (middle). Inset of the middle panel shows a magnified image of a cFos+ neuron. Note the red dot placed at the center of the soma by the automated detection algorithm. Right panel shows the binned density of the automatically detected cFos+ neurons. (C) Manually generated image mask to avoid artefact detection. Black mask covers the non-CPu structures, while red masks cover the intra-CPu artefacts (e.g., noise, vessels, etc.). (D) Distribution of intra-CPU masks at all slices along the vertical (blue) and mediolateral (red) axes. Note that the masks were distributed uniformly and covered only approximately 1% of the total CPu area. (E) Correlation of the binned BDA and cFos density values of the representative slice shown on panels A—C. The correlation coefficient of the value pairs and the significance level of the correlation are displayed above the plot. (F,G) Small (left) and large (right) magnification photomicrographs of the V2 injection site of the BDA tracer (F) and the V1 (G) of a stimulated animal. Color channels are identical to those on Fig 2. Note the large number of cFos+ neurons in both regions as a result of the visual stimulation. BDA, biotinylated dextran amine; CPu, caudate putamen; V1, primary visual cortex; V2, secondary visual cortex.
(TIF)

S2 Fig Basic electrophysiological properties and intrastriatal connectivity of the recorded CPu neurons. (A—C) Waveform and spike train characteristics of a representative PFN (A), FFN (B) and TFN (C). Top left panels: mean extracellular spike waveforms on the 8 recording sites of the corresponding electrode shank. Top-right panels: distribution of ISIs, ISI2s are aggregated. Note the high proportion of long ISIs for PFNs (red arrow). Bottom-left and right panels display the postspike suppression histograms and autocorrelograms, respectively. (D) Classification of the recorded neurons based on the features of their spike waveforms (left panel) and spike trains (right panel). (E) Proportion of the recorded PFNs (64%), FFNs (22%), TFNs (8%), and unclassified neurons (6%) among all recorded single units. CPu, caudate putamen; FFN, fast-firing neuron; ISI, interspike interval; ISI2s, interspike interval exceeding 2 s; PFN, phasically firing neuron; TFN, tonically firing neuron.
(TIF)

S3 Fig (A-D) Rate remapping of 4 example aVis+ neurons. The left panels of each example show the spatially resolved firing patterns in the uniform and striped environments (blue and red, respectively). Right panels show the details of statistical testing of the space-resolved visual modulation. The thick black lines show the real FR differences in the two environments, while the gray curves denote the label-shuffled surrogate firing pattern differences. Purple and green lines depict the “local” and “global” significance thresholds, respectively. Spatial locations breaking the global significance limits are marked with thick red lines in both the left and right panels. aVis, active visual; FR, firing rate.
(TIF)

S4 Fig FRs of the pVis+ cells in the “striped” versus “uniform” maze. The plots are showing the comparison of the normalized FRs of each pVis+ neurons in the uniform (abscissa) and in the striped maze (ordinate). Identity and regression lines are shown in black and red, respectively. Correlation coefficients and significance levels are shown above each plot. To form the FR, data points of only those consecutive trials were included in which the condition was changing from “uniform” to “striped” or vice versa. FR values were normalized by using the maximum rate during the “uniform” trials. FR, firing rate; pVis, passive visual.
(TIF)

S5 Fig Comparison of place preference of the neurons expressing place field—like activity in the “uniform” and the “striped” environment. The plots show the comparison of the normalized FRs of each place cell—like neurons in the uniform (abscissa) and in the striped maze (ordinate). Identity and regression lines are shown in black and red, respectively. Correlation coefficients, significance levels, and the coefficients of the regression lines (“a” and “b,” in which y = a × x + b) are shown above each plot. To form the FR, data points of only those consecutive trials were included in which the condition was changing from “uniform” to “striped” or vice versa. FR values were normalized by using the maximum rate during the “uniform” trials. FR, firing rate.
(TIF)

S6 Fig Response profiles of the aVis+ neurons in relation to space, context, velocity, and luminance. (A) Modulation indices of the rate remapping depend on the temporal velocity of the perceived visual motion, determined by the running velocity. Slower speeds elicited weaker decrease (i.e., weaker modulation) of the FRs. (B) Comparison of the mean running speeds in the striped and uniform environments. (C-E) Analysis of aVis+ neurons’ activity patterns in the reward zones, where the rats were not running—thus, no visual motion was perceived—but they still observed the stationary patterns of the walls. (C) The average time spent at the reward zones in trials when uniform or striped pattern were presented was similar. (D) The FRs of the aVis+ neurons were almost identical at the reward zones, with no respect to the pattern or context. (E) The distribution of the significance levels of the cell-by-cell comparison of the firing patterns depict that only a small minority of the aVis+ neurons perceived the stationary patterns as different. (F) Two representative neurons excited (left) or inhibited (right) by the global brightness increase. (G) Percent of neurons responsive to global luminance change. Note that in contrast to the aVis+ neurons, more than half of the pVis+ neurons were sensitive to brightness. (H) Response profile of a representative neuron triggered by the individual sinusoidal cycles of the grated pattern. Local luminance levels are shown above the plot; 2 full stripe cycles are shown for better visibility. Note that the stripe phase—resolved firing pattern (red) is similarly uniform as the pattern in the uniform trials (blue). (I) Mean stripe phase—resolved circular modulation indices of the aVis+ neurons were similar in the two conditions. aVis, active visual; FR, firing rate.
(TIF)

S7 Fig LFP phase coupling of the recorded neurons. Preferred LFP phases are shown as histograms for pVis−/aVis− (top row), pVis+ (second row), and aVis+ (third row) neurons separately. Bottom row shows the comparison of phase coupling strength expressed as the distribution of the phase modulation indices for the three groups of neurons (for quantification, see Fig 5C). Columns from left to right show the phase preferences for band pass—filtered LFP signals in the delta, theta, beta, low-gamma, and mid-gamma bands. For better visibility, the circular distributions are plotted twice (−180–540°) on the phase preference distribution histograms. aVis, active visual; LFP, local field potential; pVis, passive visual.
(TIF)

S8 Fig Demonstration of the concept of the novel time lag and frequency-resolved cross-correlation estimation method. (A) A demonstrative example of 2 artificial LFP signals with a phase-delayed continuous theta oscillation, 2 time-delayed discrete gamma bursts in the low- and high-gamma band, and a coincident square wave resembling a recording artefact. (B) Raw time and frequency—resolved cross-correlograms constructed from individual cross-correlograms calculated after iterative filtering for each frequency band. (C) The same time and frequency—resolved cross-correlogram after the suppression of the zero-lag correlation components. Note the emergence of 3 domains (red arrows), representing the phase-shifted theta oscillations and the gamma bursts with the appropriate time lags. Note also the lack of the artefact’s spectral profile, which dominated the raw spectrogram on panel B. LFP, local field potential.
(TIF)

## Abbreviations

a.u.: arbitrary unit
AP: anteroposterior
AuCx: auditory cortex
aVis: active visual
BDA: biotinylated dextran amine
BSA: bovine serum albumin
CPu: caudate putamen
DAPI: 4′,6-Diamidino-2-phenylindole dihydrochloride
dB: decibel
DLG: dorsolateral geniculate nucleus
DV: dorsoventral
EPSP: excitatory postsynaptic potential
FFN: fast-firing neuron
FR: firing rate
IEG: immediate early gene
ISI: interspike interval
ISI2s: interspike interval exceeding 2 s
ITCN: Image-based Tool for Counting Nuclei
LCD: liquid crystal display
LFP: local field potential
ML: mediolateral
NGS: normal goat serum
PB: phosphate buffer
PBS: phosphate-buffered saline
PBS-X: PBS containing 0.3% Triton X-100
PBS-XBSA: PBS-X containing 1% BSA
PFA: paraformaldehyde
PFN: phasically firing neuron
PSST: postspike suppression time
PV: parvalbumin
pVis: passive visual
SUA: single-unit activity
TFN: tonically firing neuron
V1: primary visual cortex
V2: secondary visual cortex
V2L: lateral secondary visual cortex
V2ML: mediolateral secondary visual cortex
V2MM: mediomedial secondary visual cortex
