# Supplementary figures and images for: Coding of self-motion-induced and self-independent visual motion in the rat dorsomedial striatum
Source: PLoS Biol. 2018 Jun 25;16(6):e2004712. doi: 10.1371/journal.pbio.2004712 (PMC6034886; doi:10.1371/journal.pbio.2004712)

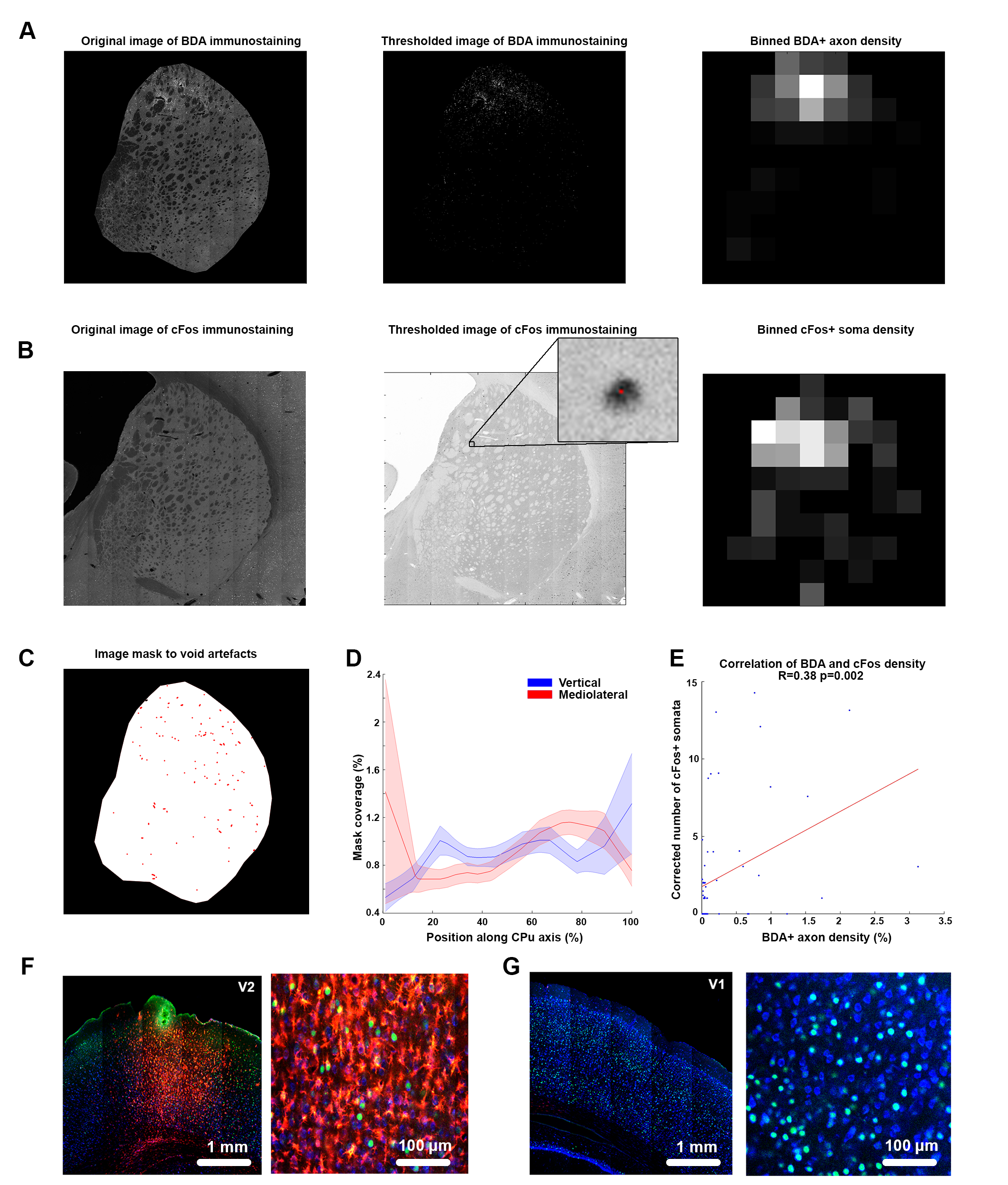

Supplement: S1 Fig — (A) Original tile-scan image of the BDA-labeled axons on a representative example slice (left). Middle panel shows the intensity-thresholded image, while right panel denotes the calculated binned axon density. (B) Original tile-scan of the cFos+ neuronal somata on the same slice (left) and its inverted version (middle). Inset of the middle panel shows a magnified image of a cFos+ neuron. Note the red dot placed at the center of the soma by the automated detection algorithm. Right panel shows the binned density of the automatically detected cFos+ neurons. (C) Manually generated image mask to avoid artefact detection. Black mask covers the non-CPu structures, while red masks cover the intra-CPu artefacts (e.g., noise, vessels, etc.). (D) Distribution of intra-CPU masks at all slices along the vertical (blue) and mediolateral (red) axes. Note that the masks were distributed uniformly and covered only approximately 1% of the total CPu area. (E) Correlation of the binned BDA and cFos density values of the representative slice shown on panels A—C. The correlation coefficient of the value pairs and the significance level of the correlation are displayed above the plot. (F,G) Small (left) and large (right) magnification photomicrographs of the V2 injection site of the BDA tracer (F) and the V1 (G) of a stimulated animal. Color channels are identical to those on Fig 2. Note the large number of cFos+ neurons in both regions as a result of the visual stimulation. BDA, biotinylated dextran amine; CPu, caudate putamen; V1, primary visual cortex; V2, secondary visual cortex. (TIF) [file pbio.2004712.s002.tif]

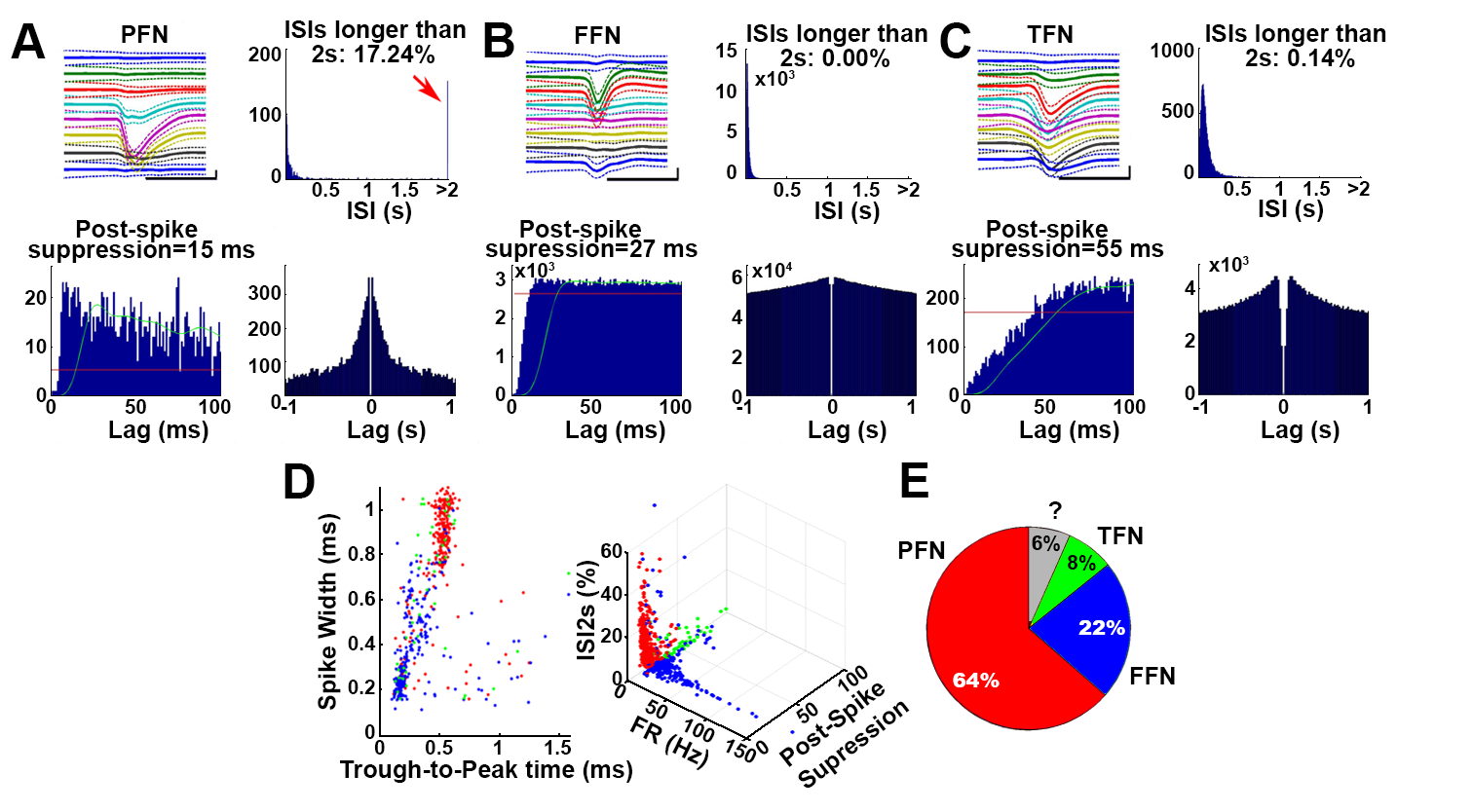

Supplement: S2 Fig — (A—C) Waveform and spike train characteristics of a representative PFN (A), FFN (B) and TFN (C). Top left panels: mean extracellular spike waveforms on the 8 recording sites of the corresponding electrode shank. Top-right panels: distribution of ISIs, ISI2s are aggregated. Note the high proportion of long ISIs for PFNs (red arrow). Bottom-left and right panels display the postspike suppression histograms and autocorrelograms, respectively. (D) Classification of the recorded neurons based on the features of their spike waveforms (left panel) and spike trains (right panel). (E) Proportion of the recorded PFNs (64%), FFNs (22%), TFNs (8%), and unclassified neurons (6%) among all recorded single units. CPu, caudate putamen; FFN, fast-firing neuron; ISI, interspike interval; ISI2s, interspike interval exceeding 2 s; PFN, phasically firing neuron; TFN, tonically firing neuron. (TIF) [file pbio.2004712.s003.tif]

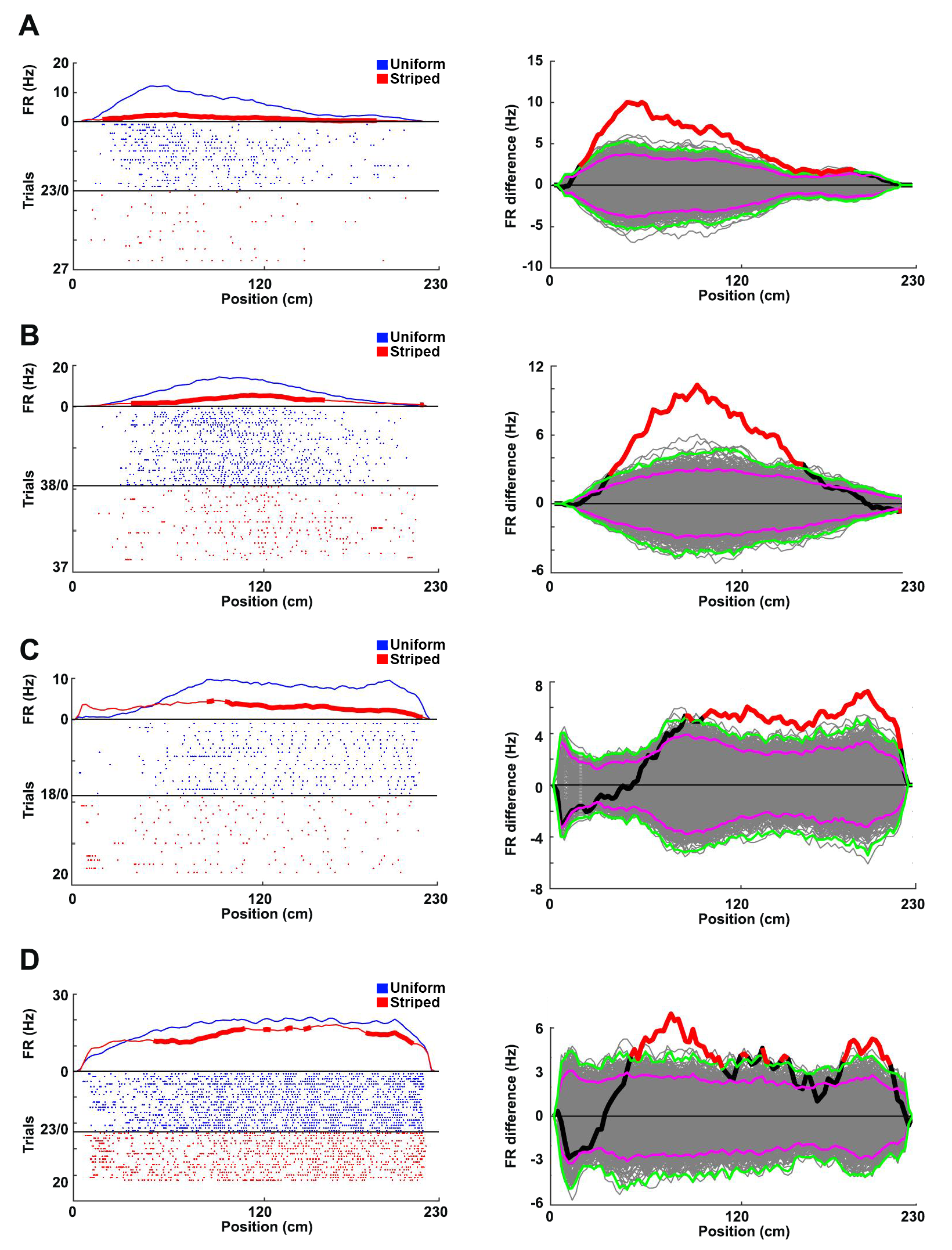

Supplement: S3 Fig — The left panels of each example show the spatially resolved firing patterns in the uniform and striped environments (blue and red, respectively). Right panels show the details of statistical testing of the space-resolved visual modulation. The thick black lines show the real FR differences in the two environments, while the gray curves denote the label-shuffled surrogate firing pattern differences. Purple and green lines depict the “local” and “global” significance thresholds, respectively. Spatial locations breaking the global significance limits are marked with thick red lines in both the left and right panels. aVis, active visual; FR, firing rate. (TIF) [file pbio.2004712.s004.tif]

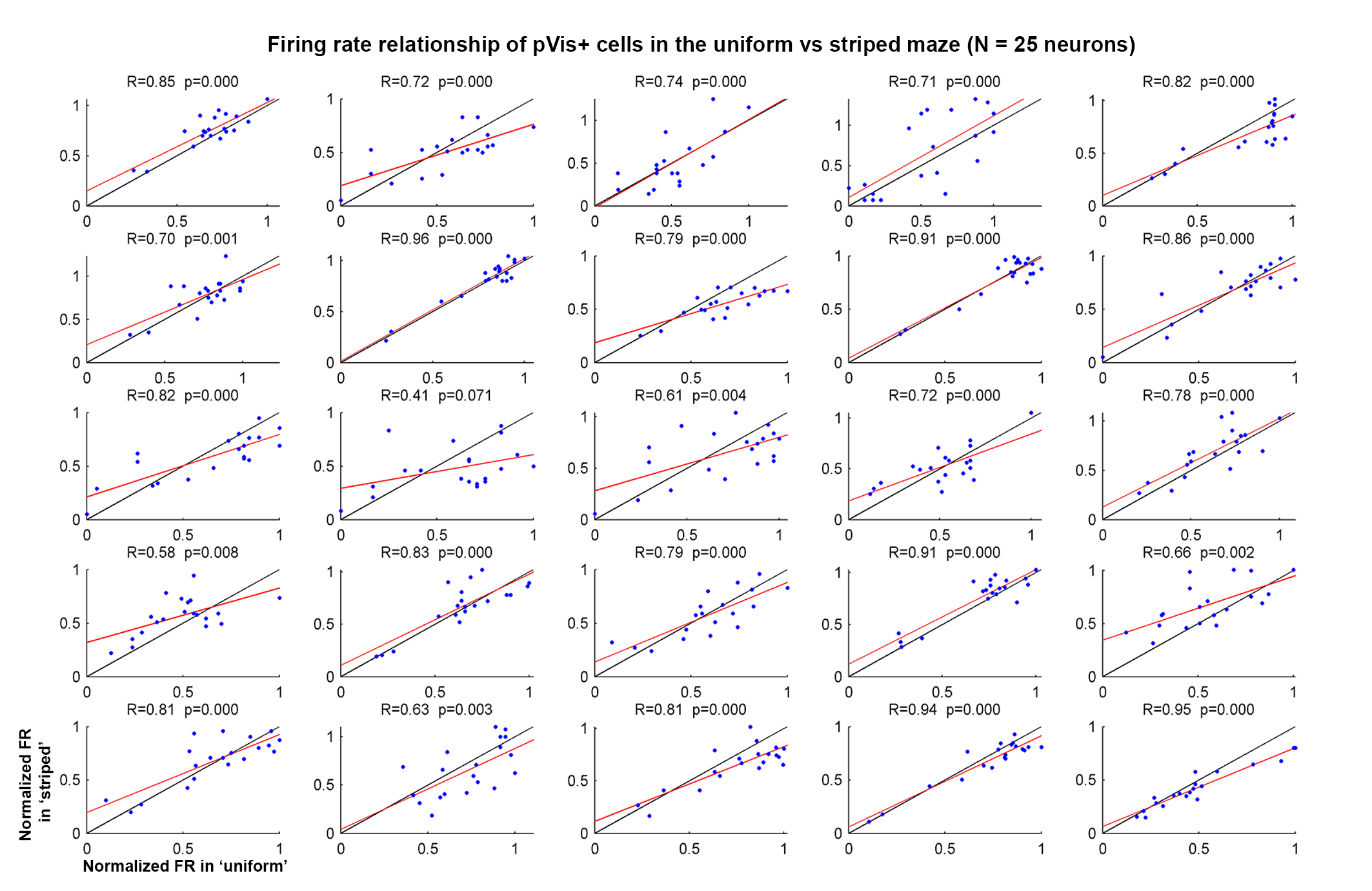

Supplement: S4 Fig — The plots are showing the comparison of the normalized FRs of each pVis+ neurons in the uniform (abscissa) and in the striped maze (ordinate). Identity and regression lines are shown in black and red, respectively. Correlation coefficients and significance levels are shown above each plot. To form the FR, data points of only those consecutive trials were included in which the condition was changing from “uniform” to “striped” or vice versa. FR values were normalized by using the maximum rate during the “uniform” trials. FR, firing rate; pVis, passive visual. (TIF) [file pbio.2004712.s005.tif]

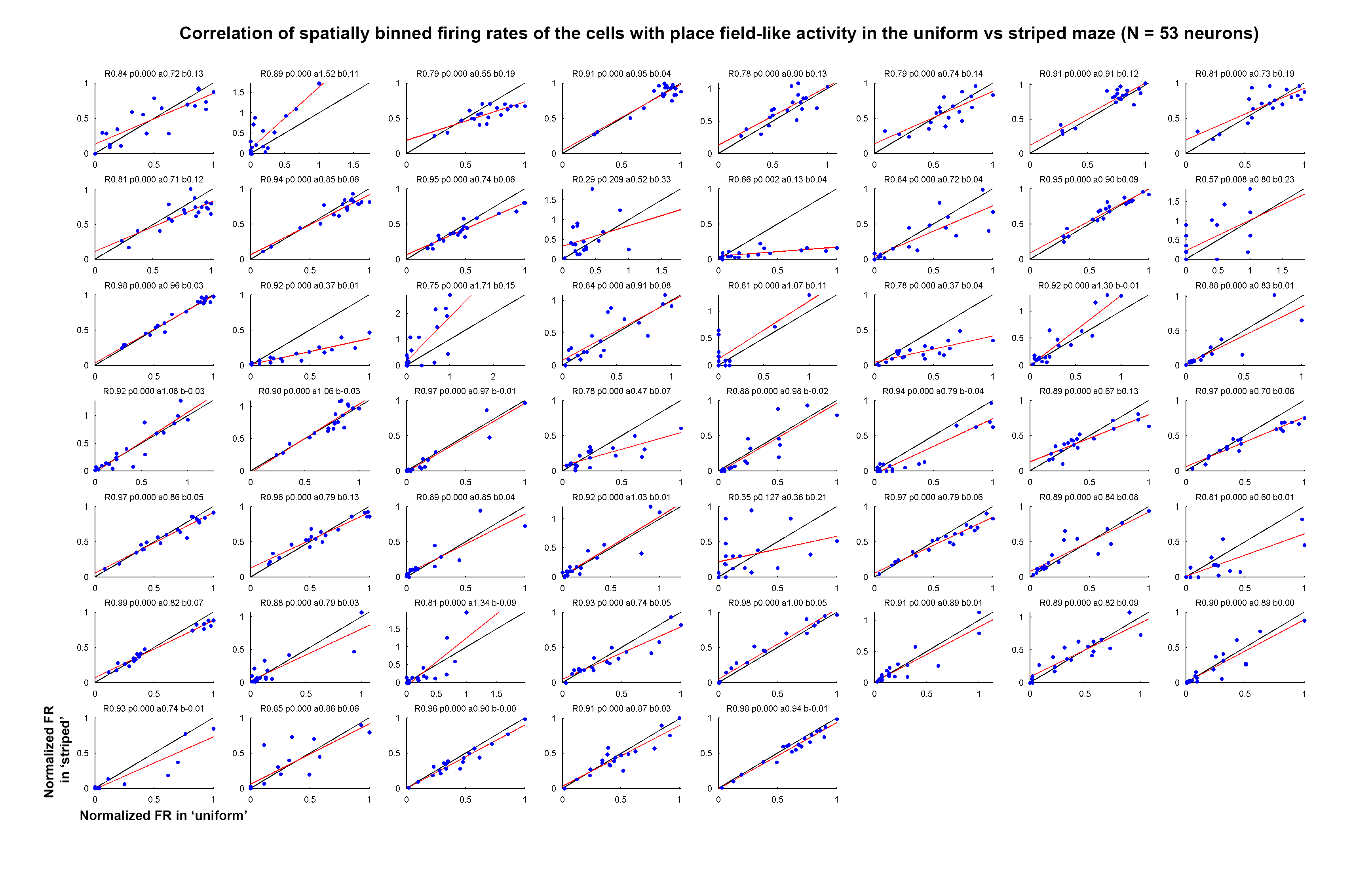

Supplement: S5 Fig — The plots show the comparison of the normalized FRs of each place cell—like neurons in the uniform (abscissa) and in the striped maze (ordinate). Identity and regression lines are shown in black and red, respectively. Correlation coefficients, significance levels, and the coefficients of the regression lines (“a” and “b,” in which y = a × x + b) are shown above each plot. To form the FR, data points of only those consecutive trials were included in which the condition was changing from “uniform” to “striped” or vice versa. FR values were normalized by using the maximum rate during the “uniform” trials. FR, firing rate. (TIF) [file pbio.2004712.s006.tif]

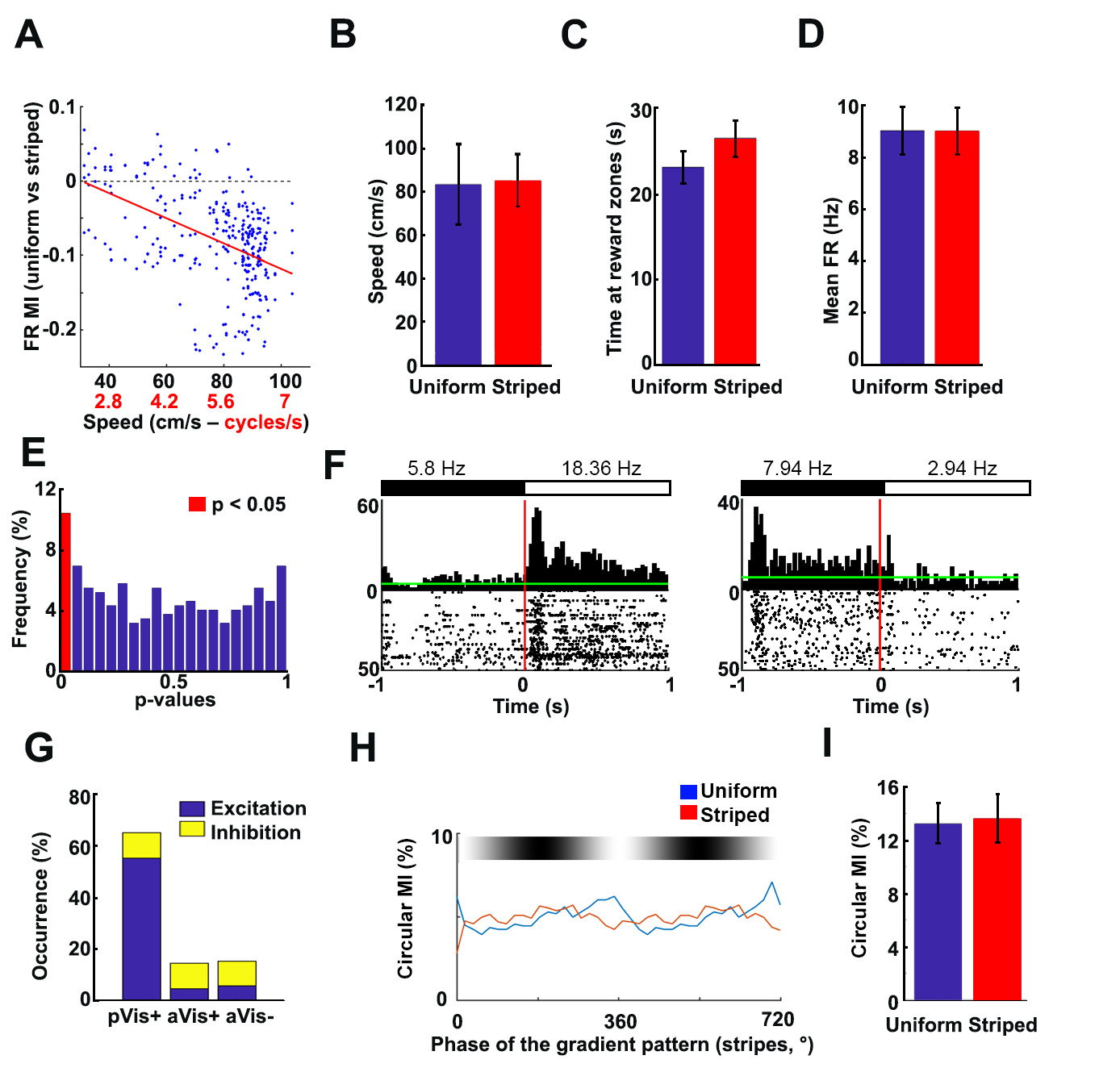

Supplement: S6 Fig — (A) Modulation indices of the rate remapping depend on the temporal velocity of the perceived visual motion, determined by the running velocity. Slower speeds elicited weaker decrease (i.e., weaker modulation) of the FRs. (B) Comparison of the mean running speeds in the striped and uniform environments. (C-E) Analysis of aVis+ neurons’ activity patterns in the reward zones, where the rats were not running—thus, no visual motion was perceived—but they still observed the stationary patterns of the walls. (C) The average time spent at the reward zones in trials when uniform or striped pattern were presented was similar. (D) The FRs of the aVis+ neurons were almost identical at the reward zones, with no respect to the pattern or context. (E) The distribution of the significance levels of the cell-by-cell comparison of the firing patterns depict that only a small minority of the aVis+ neurons perceived the stationary patterns as different. (F) Two representative neurons excited (left) or inhibited (right) by the global brightness increase. (G) Percent of neurons responsive to global luminance change. Note that in contrast to the aVis+ neurons, more than half of the pVis+ neurons were sensitive to brightness. (H) Response profile of a representative neuron triggered by the individual sinusoidal cycles of the grated pattern. Local luminance levels are shown above the plot; 2 full stripe cycles are shown for better visibility. Note that the stripe phase—resolved firing pattern (red) is similarly uniform as the pattern in the uniform trials (blue). (I) Mean stripe phase—resolved circular modulation indices of the aVis+ neurons were similar in the two conditions. aVis, active visual; FR, firing rate. (TIF) [file pbio.2004712.s007.tif]

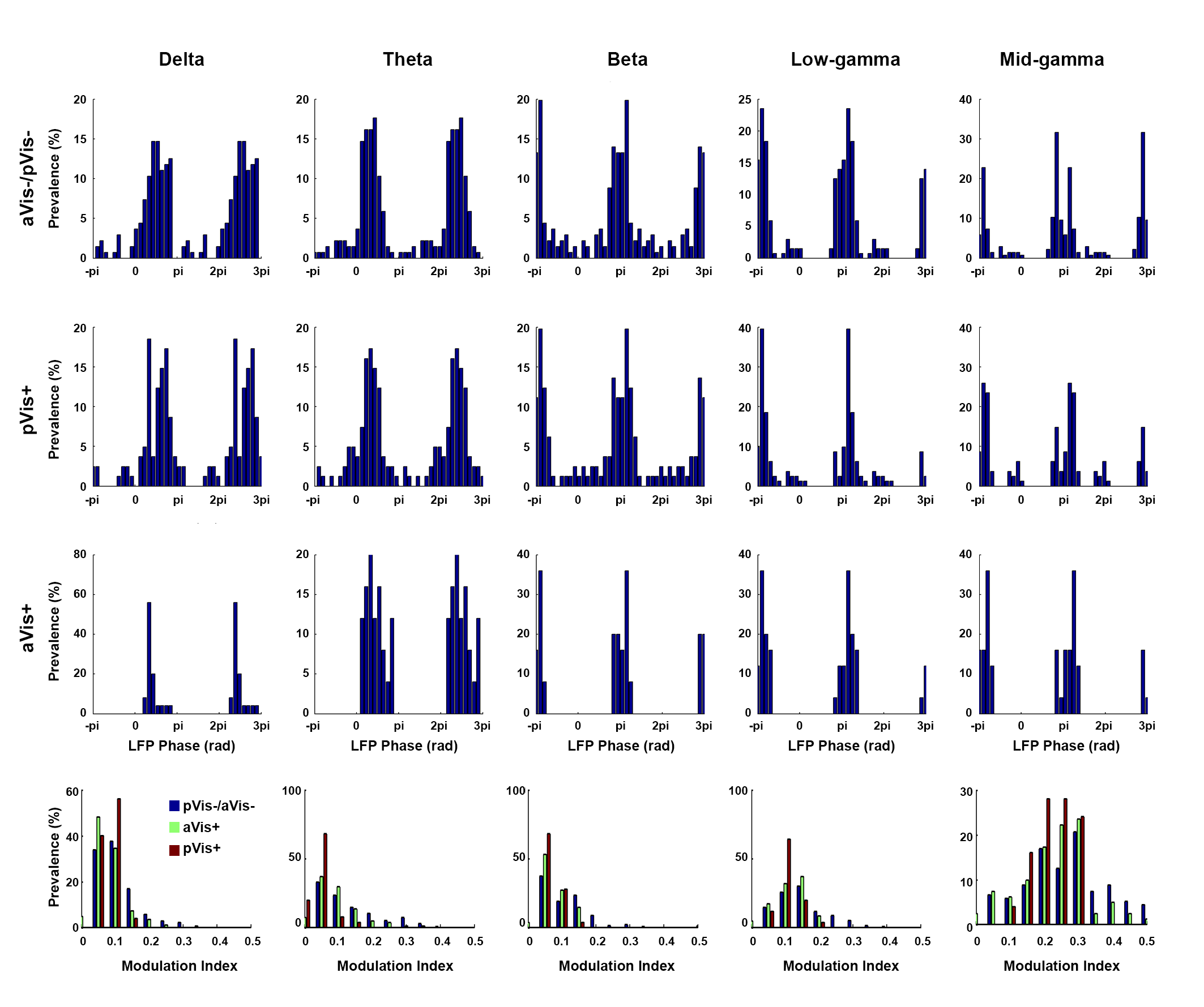

Supplement: S7 Fig — Preferred LFP phases are shown as histograms for pVis−/aVis− (top row), pVis+ (second row), and aVis+ (third row) neurons separately. Bottom row shows the comparison of phase coupling strength expressed as the distribution of the phase modulation indices for the three groups of neurons (for quantification, see Fig 5C). Columns from left to right show the phase preferences for band pass—filtered LFP signals in the delta, theta, beta, low-gamma, and mid-gamma bands. For better visibility, the circular distributions are plotted twice (−180–540°) on the phase preference distribution histograms. aVis, active visual; LFP, local field potential; pVis, passive visual. (TIF) [file pbio.2004712.s008.tif]

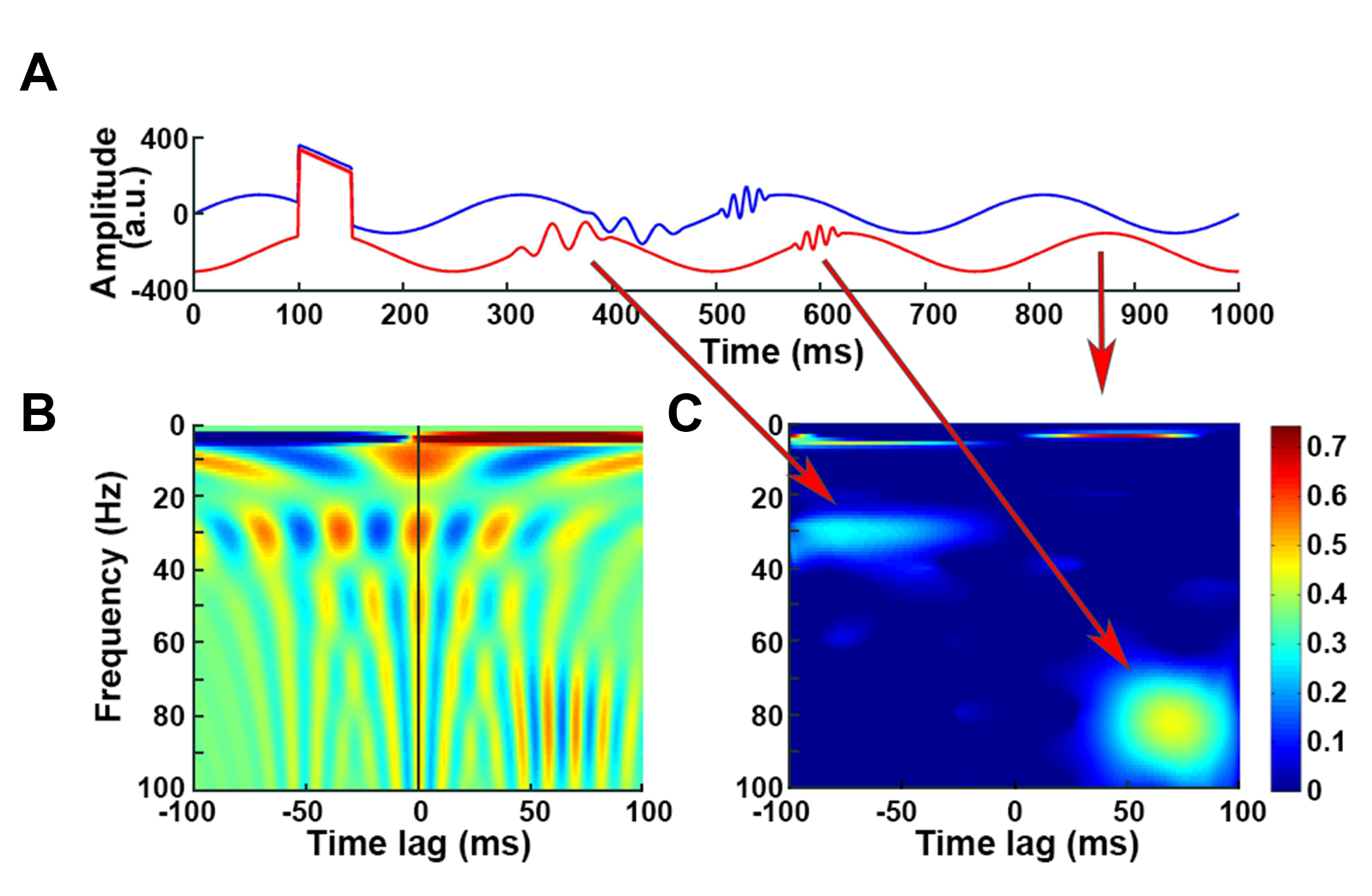

Supplement: S8 Fig — (A) A demonstrative example of 2 artificial LFP signals with a phase-delayed continuous theta oscillation, 2 time-delayed discrete gamma bursts in the low- and high-gamma band, and a coincident square wave resembling a recording artefact. (B) Raw time and frequency—resolved cross-correlograms constructed from individual cross-correlograms calculated after iterative filtering for each frequency band. (C) The same time and frequency—resolved cross-correlogram after the suppression of the zero-lag correlation components. Note the emergence of 3 domains (red arrows), representing the phase-shifted theta oscillations and the gamma bursts with the appropriate time lags. Note also the lack of the artefact’s spectral profile, which dominated the raw spectrogram on panel B. LFP, local field potential. (TIF) [file pbio.2004712.s009.tif]
